# A single nucleotide mutation of *BnaC05.POLIB* creates yellow-white chimeric flower in *Brassica napus*

**DOI:** 10.1093/hr/uhaf276

**Published:** 2026-01-01

**Authors:** Rui Xia, Lin Chen, Pengfei Wang, Baoying Huang, Baoling Liang, Shengzhe Lin, Guangsheng Yang, Dengfeng Hong

**Affiliations:** National Key Laboratory of Crop Genetic Improvement, College of Plant Science and Technology, Huazhong Agricultural University, Wuhan 430070, China; National Key Laboratory of Crop Genetic Improvement, College of Plant Science and Technology, Huazhong Agricultural University, Wuhan 430070, China; National Key Laboratory of Crop Genetic Improvement, College of Plant Science and Technology, Huazhong Agricultural University, Wuhan 430070, China; National Key Laboratory of Crop Genetic Improvement, College of Plant Science and Technology, Huazhong Agricultural University, Wuhan 430070, China; National Key Laboratory of Crop Genetic Improvement, College of Plant Science and Technology, Huazhong Agricultural University, Wuhan 430070, China; National Key Laboratory of Crop Genetic Improvement, College of Plant Science and Technology, Huazhong Agricultural University, Wuhan 430070, China; National Key Laboratory of Crop Genetic Improvement, College of Plant Science and Technology, Huazhong Agricultural University, Wuhan 430070, China; Hubei Hongshan Laboratory, Wuhan 430070, China; National Key Laboratory of Crop Genetic Improvement, College of Plant Science and Technology, Huazhong Agricultural University, Wuhan 430070, China; Hubei Hongshan Laboratory, Wuhan 430070, China

## Abstract

Flower color is a key trait influencing insect pollination and ornamental value, yet the molecular mechanisms underlying heterozygous flower color remain unclear. In this study, we identified the creation of a yellow-white chimeric flower (*cf*) mutation in *Brassica napus*, characterized as the coexistence of yellow and white colors on petals of the same flower. Genetic analysis revealed that chimeric flower formation is controlled by a completely dominant gene. Map-based cloning, transgenic complementation, and CRISPR/Cas9 experiments consistently confirmed that BnaC05G0385300ZS on chromosome C05 is the causal gene of *CF*, which encodes a plastid DNA polymerase IB (*BnaC05.POLIB*). A G-to-A mutation in the seventh exon results in a D742N substitution, which disrupts Mg^2+^ binding and impairs polymerase activity. This leads to a reduced plastid genome copy number, decreased chromoplast formation, and aberrant carotenoid accumulation, ultimately resulting in the chimeric phenotype in a dosage-dependent manner. These findings reveal a novel role for *BnaC05.POLIB* in petal color patterning and provide a strategy for breeding ornamental plants with heterozygous flowers.

## Introduction

Flower color is a critical trait in plants, serving not only as an esthetic feature but also as a key factor in environmental adaptation, pollination, and genetic diversity [1, 2]. Flower color in angiosperms is highly diverse, exhibiting a range of hues and forming distinctive color patterns, with various parts of the petals displaying different color arrangements, referred to as floral patches [3, 4]. The formation of these patterns influences pollinator preferences and facilitates successful pollination [5, 6]. Additionally, floral spots in certain plants play a significant role in deterring herbivorous insects [7]. In the contemporary floricultural industry, floral spots contribute to the enhancement of color diversity and, consequently, increase the commercial value of flowers [8–10]. Thus, understanding the mechanisms behind flower spot formation is crucial for advancing research in this area.

In nature, plant flower color is primarily determined by three major pigment classes: flavonoids, carotenoids, and betalains [11–13]. Flavonoids are key to yellow, red, and blue hues, while carotenoids drive yellow to orange variations. Betalains, found mainly in the Caryophyllales order, produce yellow or red flowers [14]. Differential pigment accumulation within petals creates unique patterns, as seen in various species like Peony ‘Erqiao’, *Clarkia*, *Mimulus*, and *Helianthus argophyllus* [15–18]. For example, in *Nigella orientalis*, chlorophyll accumulates early, carotenoids midstage, and anthocyanins late, forming complex yellow petals with green spots, fuchsia stripes, and mottling [19]. Changes in petal cell pH can also induce mottling in otherwise solid-colored flowers [20]. In Petunia, *PH5* (P-type H^+^-ATPase 5) and *AN1* (ANTHOCYANIN1) facilitate intravacuolar acidification, leading to mottled mutants [21]. Meanwhile, competition between structural genes encoding enzymes that target the same substrate, such as FLS (flavonoid 3′ hydroxylase) and DFR2 (dihydrokaempferol 2), which competitively bind to naringenin in the flavonoid pathway, drives pigment synthesis and flower color variation [22].

*Brassica napus* (rapeseed), a significant oilseed crop, has diverse uses [23], and its flower color contributes to tourism and economic development [24]. Traditional *B. napus* flowers are mostly pure yellow, but recent studies have reported pure white, orange, and red flowers [25]. Carotenoids primarily produce yellow to red hues. In rapeseed, yellow petal coloration results from carotenoid accumulation. Conversely, the white-flowered phenotype of *B. napus* line 2127 is correlated with the *BnaC3.CCD4* gene, which encodes carotenoid cleavage dioxygenase 4 (CCD4), an enzyme involved in carotenoid degradation. The formation of yellow petals depends on the accumulation of carotenoids. Overexpression of *BnaC3.CCD4* (encoding carotenoid cleavage dioxygenase 4) enhances carotenoid degradation, ultimately yielding white petals. The insertion of a transposon into the *BnaC3.CCD4* gene of yellow-flowered rapeseed led to functional inactivation, resulting in the accumulation of carotenoids in the petals and, consequently, the yellow coloration [26]. Orange-flowered rapeseed is regulated by a pair of zeaxanthin epoxidase genes, *BnaA09.ZEP* and *BnaC09.ZEP*; and mutations in these alleles lead to carotenoid accumulation in the petals [27]. Furthermore, functional differentiation of BnaZEPs occurs in *B. napus*, with *BnaA07.ZEP* and *BnaC07.ZEP* primarily involved in the synthesis of carotenoids and ABA in leaves, influencing the drought tolerance of *B. napus* [28]. An enhancer insertion in the promoter region of *BnaA07.PAP2* activates its expression, leading to the accumulation of anthocyanins in the petals and causing the apricot-red flower color [29]. However, there have been no reports on the discovery of accessions with chimeric flower color in *B. napus* or even in Brassiceae plants. Therefore, studying the genetic basis of chimeric flower formation in *B. napus* and uncovering the molecular mechanisms of chimerism will not only provide a theoretical foundation for breeding colorful-flowered rapeseed but also offer a molecular framework for flowering plants.

In plants, the nucleus-encoded DNA polymerase POLIB plays an essential role in organellar genome maintenance. In *Arabidopsis*, *POLIB* is dual-targeted to mitochondria and chloroplasts and is involved in both DNA replication and repair, with *polib* mutants showing reduced organellar DNA copy number and increased sensitivity to DNA damage [30]. It acts synergistically with plastid Whirly proteins to suppress microhomology-mediated recombination and maintain genome stability [31]. Functional divergence exists among species: while dicots like *Arabidopsis* and tobacco possess two partially redundant polymerases, monocots such as maize rely on a single enzyme for plastid DNA replication, with POLIB orthologs contributing specifically to mitochondrial genome integrity [32]. These findings underscore the pivotal and species-specific roles of POLIB in safeguarding organellar DNA.

Here we report a *B. napus* mutant obtained by ethyl methanesulfone (EMS) mutagenesis that exhibits stably inherited yellow-white chimeric flower (*cf*), and revealed the genetic and molecular basis of chimeric flower formation in *B. napus* by using diverse approaches including map-based cloning, genetic transformation, metabolomics, cytological investigation, and transcriptome analysis. These results provide novel insights into the molecular mechanism on floral spot formation and emphasize the significance of copy number alteration of plastids genome in plant development.

## Results

### *cf* displays a typical yellow-white chimeric flower and is controlled by a dominant locus

**Figure 1:**
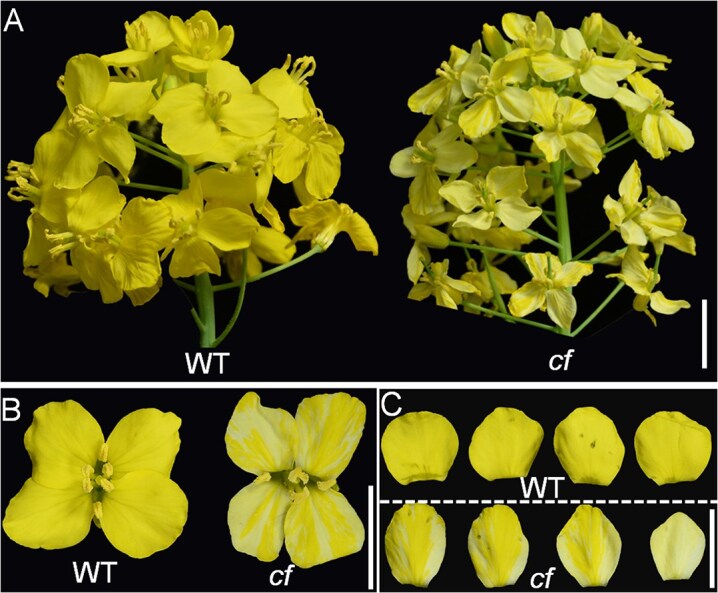
Flower morphology of the parental lines in *B. napus*. (A) The inflorescence of WT (left) and *cf* (right). Bar, 1 cm. (B) The flower color of WT (left) and *cf* (right). Bar, 1 cm. (C) The petals of WT (up) and *cf* (down) flower. Bar, 1 cm.

From an EMS-mutagenized M₁ population of *B. napus*, we identified a mutant exhibiting yellow-white chimeric flowers. Compared to the uniformly yellow petals of wild-type (WT) flowers, the *cf* mutant petals displayed obvious variegation, with distinct yellow and lighter (white-like) streaks (Fig. 1A), indicating a disrupted pigment distribution. Moreover, the position and area proportion of white and yellow parts in *cf* seems random in various petals, but generally showing a white and yellow interspersed striped distribution pattern (Fig. 1B and C). Additionally, the leaf epidermis of mutant monocultures at later developmental stages is characterized by ‘water-soaked’ mottling (Fig. S1A). In terms of other agronomic traits, the *cf* mutant showed no significant difference in thousand-seed weight compared to the WT; however, both silique length and the number of seeds per silique were decreased (Fig. S1B–E). These results suggest that the coloring pattern of chimeric flowers may be different from that of normal pure color flowers.

To verify the genetic stability of the *cf* mutant, we successively self-pollinated the *cf* mutant. The results revealed consistent segregation of yellow and chimeric flowers in its progeny, indicating embryonic lethality in the homozygous mutant. Statistical analysis of flower color segregation in the M_5_ population revealed 704 chimeric flowers and 358 yellow-flowered individuals out of 1062 total plants. The segregation ratio of yellow and white chimeric flowers was consistent with the theoretical 1:2 ratio, as determined by a chi-square test (χ^2^ = 0.052, *P* > 0.05). In crossing experiments between the *cf* mutant and the WT line Xiaoyun, the petals of F_1_ plants exhibited a 1:1 segregation ratio of yellow and chimeric flowers (Table 1, Fig. S2). These results suggest that the yellow-white flower trait in *B. napus* is controlled by a single dominant nuclear gene, with a lethal effect in the homozygous *cf* chimera. This locus was designated as *CF*.

**Table 1:** Genetic analysis of chimeric flower in *B*. *napus*

Generations	Population	Total plants	Yellow petal	Yellow and white petal	Expected ratio	*χ* ^2^	*P*-value
M_5_	R75–1009	166	58	108	1:2	0.127	*P* > 0.05
	R75–1209	189	63	126	1:2	0.006	*P* > 0.05
	R75–1139	145	55	90	1:2	1.180	*P* > 0.05
	R75–1145	562	182	380	1:2	0.187	*P* > 0.05
F_2_	*cf*-xiaoyun	506	176	330	1:2	0.415	*P* > 0.05

### MUTMAP analysis anchors the candidate region of *cf* on chromosome C05

**Figure 2:**
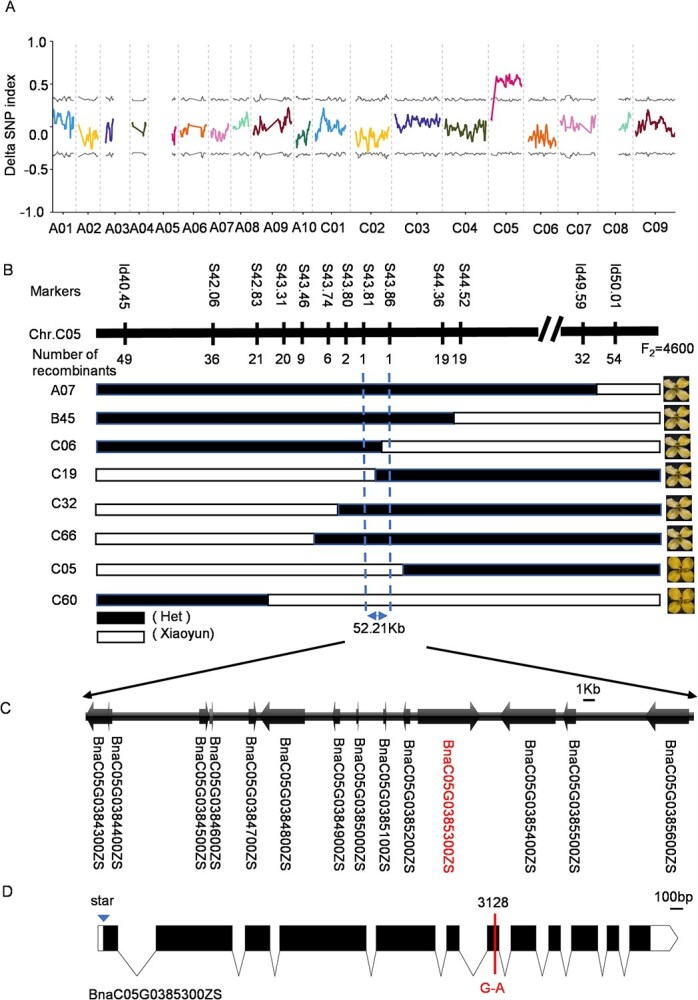
Map-based cloning of CF site. (A) The Δ(SNP-index) value on chromosomes in reference genome ‘ZS11’ through MutMap. Only Chromosome C05 had a peak above the threshold. (B) The CF locus was delimited to a 52.21-kb interval between two flanking markers, S43.81 and S43.86, using eight recombinant plants with extreme phenotypes from 4600 F_2_ individuals. (C) Annotated genes in the CF locus. Bar = 1 kb. The red letter indicates the most likely candidate gene for CF. (D) Gene structure and mutation sites of *BnaC05.POLIB*, including nucleotide substitutions in WT and *cf*. A substitution mutation (G to A) was identified at nucleotide position 3128. Bar, 100 bp.

To identify candidate quantitative trait loci (QTLs) associated with chimeric flower color, we performed MutMap analysis using genome resequencing data. Thirty yellow-flowered individuals and 30 yellow-white chimeric-flowered individuals were randomly selected from 400 M_5_ individuals, and H-pools and HB-pools were constructed, respectively. Sequencing of the two pools (H-pool and HB-pool) was performed on the Illumina HiSeq™ PE150 platform. Following the removal of low-quality and short reads, the H-pool yielded 26.8 Gb of clean data, while the HB-pool produced 26.9 Gb (Table S1). Subsequently, filtered reads from each sample were aligned against the rapeseed reference genome (ZS11). This alignment achieved localization rates between 97.54% and 98.31%, with an average sequencing depth averaging 30× (Fig. S3). Single nucleotide polymorphism (SNP) indices (SNP-index) were calculated for each SNP position in both pools to display the SNP distribution across each chromosome in *B. napus* using a sliding window approach. The ΔSNP-index was derived from the difference in SNP indices between both pools. Using the ΔSNP-index for visual mapping, only the mean ΔSNP value in the C05 chromosome interval exceeded 0.5, surpassing the 95% confidence interval threshold for the entire genome. Finally, we identified a candidate region of 40.7 Mb on chromosome C05 (Fig. 2A).

Given the broad candidate intervals and the difficulty in detecting SNP markers, we generated new F_2_ populations (*cf* × Xiaoyun) to fine-map the CF locus. To confirm and refine the genetic intervals identified from the MutMap analysis, fine localization was performed using INDEL and SNP markers based on the initial candidate regions. Two INDEL markers were developed at the starting position of the candidate region, along with 10 SNP markers within the interval. Recombination screening and phenotype–genotype association analysis were performed using 4600 F_2_ individuals. The results showed that eight recombinants within the interval exhibited chimeric flower phenotypes, confirming that the previously identified genetic interval was associated with chimeric flower color. Among them, C06 and C19 were genotyped differently at markers S43.81 and S43.86, and both exhibited chimeric flower phenotypes. The target region was narrowed to 52.21 kb (between markers S43.81 and S43.86) via recombinant-based phenotype–genotype association analysis. This critical interval was demarcated by two defining recombinants. (Fig. 2B).

According to the ZS11 reference genome annotation, a total of 14 genes were annotated within the 52-kb candidate interval (Fig. 2C, Table S2). To further validate the candidate genes, MutMap sequencing data were used to analyze the variant sites within the 52-kb interval in the WT and *cf* mutant. Only one single-base variant (G3128A) in *cf* was identified in the seventh exon of BnaC05G0385300ZS, resulting in a D742N substitution at the protein level (Fig. 2D). BnaC05G0385300ZS is predicted to encode a B-type DNA polymerase I (POLIB). Sequence alignment analysis revealed that it has two copies in *B. napus*, and their protein sequences exhibit high similarity, indicating that they may possess identical functions (Fig. S4, Table S3). Multiple protein sequence alignment revealed that the affected residue is highly conserved across crop plants (Fig. S5), suggesting that the D742N substitution may be responsible for the chimeric flower color in the *cf* mutant.

### Functional verification by transgenic experiments

**Figure 3:**
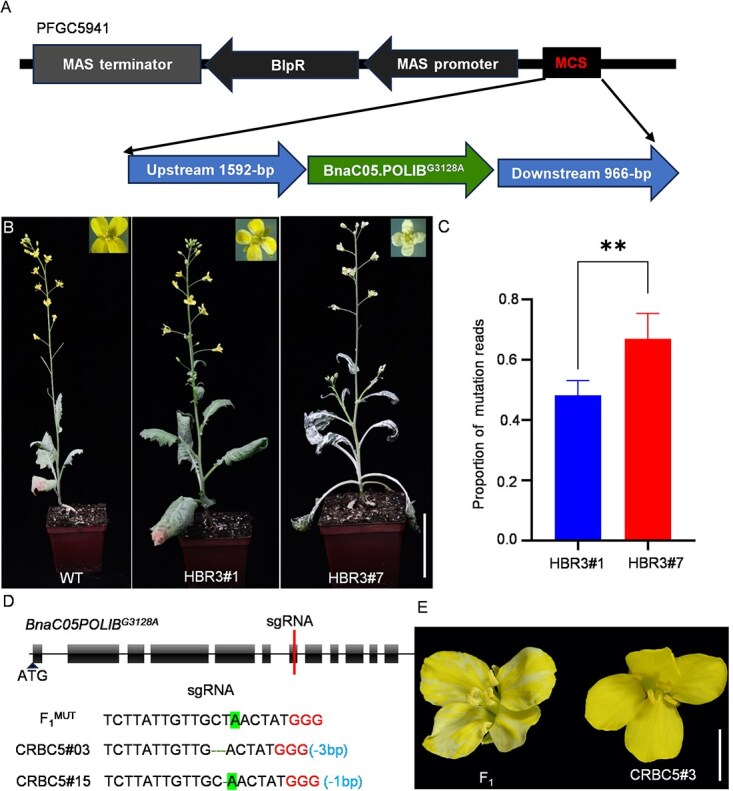
Verification of genetic complementarity and gene editing. (A) Vector construction schematic: the mutated *BnaC05.POLIB* gene fragment—comprising a 1592-bp promoter, coding sequence, and 966-bp downstream region—was cloned into the PFGC5941 backbone vector. (B) Phenotypic characterization of transgenic rapeseed plants carrying *BnaC05.POLIB^G3128A^*. Five independent transgenic lines were generated, with two representative lines selected for phenotypic analysis. HBR3#1 showed chimeric flower, HBR3#7 showed a more pronounced whole-plant albino phenotype. Bar, 10 cm. (C) Comparison of the proportion of mutant reads (G–A) in the HBR3#1 and HBR3#7 strains using RNA-Seq. ^**^P < 0.01. (D) Positions of sgRNA targeted to *BnaC05.POLIB^G3128A^*, which was special targeted to mutation site (base highlighted), The last three bases (GGG) are PAM sequence, two representative lines were performed. (E) Phenotype of representative edited line, the edited plant shows normal yellow flower color. Bar, 1 cm.

To determine whether the nonsynonymous mutation in *BnaC05.POLIB* was responsible for *cf* floral chimerism, we expressed the mutant *BnaC05.POLIB* in the Xiaoyun parent under its native promoter (Fig. 3A). Ultimately, five positive transgenic lines were obtained (Fig. S6; Table S4). The T_1_ generation of independent transgenic lines exhibited yellow and chimeric flower coloration, with phenotypes similar to *cf* when compared to Xiaoyun (Fig. 3B). Furthermore, the entire plant exhibited severe color chimerism in both the HBR3#7 and HBR3#15 lines. Expression analysis revealed that the proportion of mutant reads was significantly higher in strain HBR3#7 than in HBR3#1, and the degree of floral color chimerism increased with higher expression of *BnaC05.POLIB^G3128A^* (Fig. 3C). Additionally, we employed CRISPR-mediated gene editing to target the mutation site in the (*cf* × Xiaoyun) hybrid plants by introducing an sgRNA specific to *BnaC05.POLIB* (Fig. 3D). In the resulting T_1_ generation, plants carrying different mutation genotypes but without the original EMS-induced G3128A mutation in *BnaC05.POLIB* restored the homozygous yellow flower color. This result indicates that the G3128A mutation is critical for chimeric flower formation. Moreover, the novel mutation genotypes of *BnaC05.POLIB* failed to alter flower color, suggesting that it may be functionally redundant in *B. napus* (Fig. 3E). These transgenic assays confirm that *BnaC05.POLIB* plays a role in controlling flower color in rapeseed and that the D742N substitution is essential for its molecular function.

### D742N mutation may disrupt the canonical polymerase catalytic activity of *BnaC05.POLIB*

**Figure 4:**
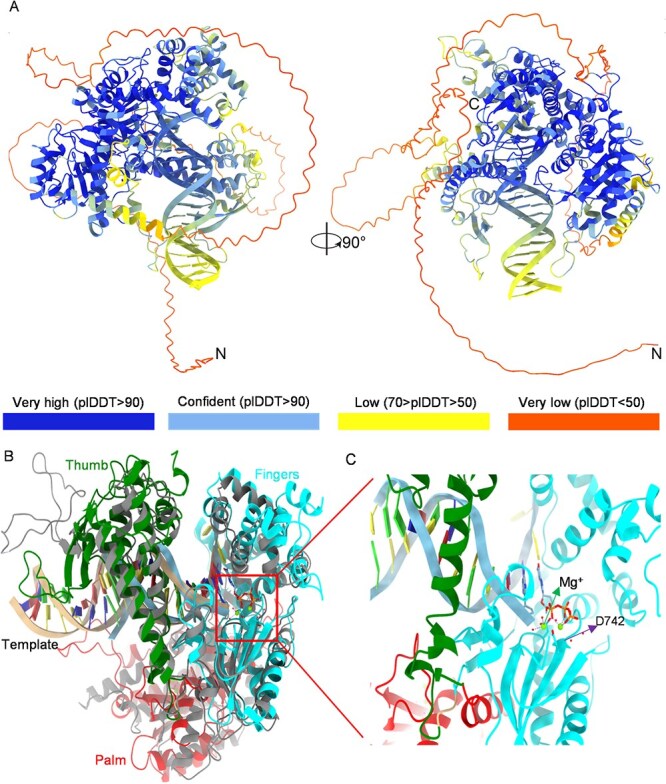
Structural and functional analysis of BnaC05POLIB protein. (A) Predicted structure of the complex, visualized using ChimeraX. The N-terminal and C-terminal ends of the proteins are labeled. Residues are colored according to their pLDDT score, indicating the confidence in the predicted structure: very high (pLDDT >90) in dark blue, confident (90 > pLDDT >70) in light blue, low (70 > pLDDT >50) in yellow, and very low (pLDDT <50) in red. (B) A comparison with the overall structure of phage T7 DNA polymerase, highlighting the high degree of structural similarity between the two enzymes. The complex adopts a classic polymerase architecture, with the thumb, palm, and fingers domains colored in green, red, and cyan, respectively. The DNA template and primer strands are shown in orange. (C) Close-up view of the active site of BnaC05-POLIB, showing the template DNA, primer strand, incoming dGTP, and two catalytic Mg ions. The bacteriophage T7 DNA polymerase is hidden for clarity.

To answer how the D742N mutation in *BnaC05.POLIB* confers a dominant hereditary effect on flower color, we attempted to establish the relationship between the 3D structural change of *BnaC05.POLIB* and its catalytic activity. AlphaFold2 has been recognized for its revolutionary breakthrough in protein structure prediction, with its reliability and accuracy widely validated [33]. Here we employed AlphaFold3, an advanced deep learning-based method, to predict and analyze the structure of the *BnaC05.POLIB*-DNA complex in detail. Using AlphaFold3’s sophisticated prediction framework, we obtained five protein structure models with varying prediction confidence, all exhibiting strong convergence, indicative of high overall model quality. Notably, the highest scoring model (Fig. 4A) achieved an ipTM score of 0.91 and a pTM score of 0.76, with most regions exhibiting pLDDT (predicted Local Distance Difference Test) scores >90%, confirming the high reliability of the predicted structure. Structural analysis of the BnaC05-POLIB-DNA complex revealed that the complex exhibits classical polymerase structural features, comprising three distinct domains: the thumb, palm, and fingers domain (Fig. S6A).

We then performed a detailed structural analysis of the BnaC05-POLIB-DNA complex and compared it with the classical phage T7 DNA polymerase [34]. The results revealed high structural similarity between the two enzymes, with a root mean square deviation (RMSD) of 1.3 Å, further supporting that BnaC05-POLIB adopts the classical polymerase architecture comprising three domains: thumb, palm, and fingers (Fig. 4B). Moreover, the active sites of both enzymes are highly conserved, suggesting similar catalytic mechanisms. Structural analysis identified two magnesium ions in the active center; their interactions with surrounding amino acids are essential for DNA polymerase activity. The SNP generated by EMS mutagenesis in this study corresponds to amino acid position D742, located precisely at the Mg^2+^ binding site (Fig. 4C). This residue plays an essential role in the bimetallic ion mechanism of DNA polymerase catalysis by coordinating one of the two required Mg^2+^ ions. This coordination facilitates proper positioning of the incoming deoxyribonucleoside triphosphate (dNTP) and stabilizes the transition state during nucleophilic attack on the dNTP’s α-phosphate group. Mutation of D742N would significantly impair DNA polymerase activity (Fig. S7B). Substituting the negatively charged aspartic acid (D) with polar but uncharged asparagine (N) is expected to weaken Mg^2+^ coordination, potentially reducing catalytic efficiency or abolishing activity entirely.

### Mutation of *BnaC05.POLIB* interfered with carotenoid synthesis and accumulation by reduced plastid genome copy number in petals

**Figure 5:**
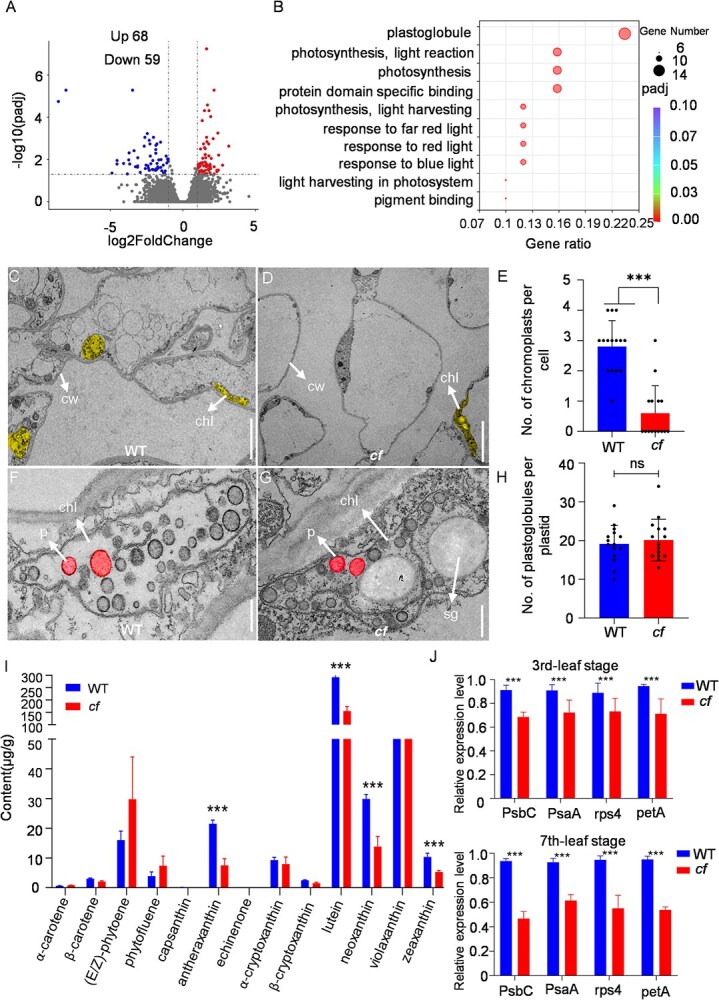
*BnaC05POLIB^G3128A^* influences the formation of chimeric flower in *B. napus.* (A) Volcano plot visualizing petal transcriptome differences between WT and *cf* mutants. (B) GO annotation analysis, showing the top 10 pathways. (C–E) Observation of cellular structure. (C) Observation of WT organelle; (D) Observation of *cf* organelle; (E) statistics on the number of chromoplast between WT and *cf* per cell. cw, cell wall; chr, chromoplast. Bar, 50 μm. The error bar is used to indicate the standard error. ^***^P < 0.001. (F–H) Observation of structure of chromoplast. (F) Observation of WT chromoplast. Bar, 10 μm. (G) Observation of *cf* chromoplast. Bar, 10 μm. (H) Statistics on the number of plastoglobules between WT and *cf* per chromoplast. P, plastoglobule; sg, starch grains. (I) Carotenoid components in petals of WT and *cf*. The error bar is used to indicate the standard error. ^***^P < 0.001. The horizontal axis represents the category of carotenoid-like pigments in the petals. (J) Relative level of ctDNA copy number at third-leaf stage and seventh-leaf stage. The horizontal axis represents unique genes in the chloroplasts.

To elucidate the molecular basis underlying the involvement of *BnaC05.POLIB* in chimeric flower color formation, yellow and chimeric flowers isolated from the M_5_ progeny were subjected to transcriptome analysis. Analysis of petals revealed 68 upregulated and 59 downregulated genes (Fig. 5A). All differentially expressed genes (DEGs) were annotated using the Gene Ontology (GO) and Kyoto Encyclopedia of Genes and Genomes (KEGG) databases. The DEGs between the two petals were significantly enriched in the main GO terms: ‘plastoglobule’ and ‘photosynthesis’, suggesting that both photosynthesis and chromoplast development were impaired in the petals (Fig. 5B, Fig. S8). These findings indicate that *BnaC05.POLIB* influences plastid development in petals, thereby affecting carotenoid synthesis and accumulation, which ultimately contributes to the formation of chimeric flower colors. Therefore, we utilized transmission electron microscopy to observe the petal cells of WT and *cf*. The results showed that the cell size and shape appeared normal, intracellular organelles were uniformly distributed, and distinct chromoplast were visible in the petal cells of WT (Fig. 5C). Additionally, numerous plastoglobules were aggregated within chromoplasts in WT (Fig. 5F), indicating normal accumulation and storage of carotenoids in WT petals. In contrast, the *cf* mutant exhibited expanded cellular spaces, reduced cytoplasm in some cells, and only a few cells with normal chromoplast development; most cells lacked chromoplasts entirely (Fig. 5D–E). Further examination of plastoglobule development in both materials revealed no obvious abnormalities in the number or morphology of plastoglobule in cells containing chromoplasts (Fig. 5F–H), thus the petals still show the normal yellow part of the flower. These results suggest that *BnaC05.POLIB* influences the number of chromoplast, consistent with the above results from transcriptome analysis.

Subsequently, we analyzed the composition and content of carotenoids in the petals of both mutant and WT flowers. The results showed that the total carotenoid content was 443.94 ± 24.03 μg/g in WT line 7–5, compared to 282.08 ± 28.59 μg/g in the yellow-white chimeric line *cf* (Table S4). Specifically, the differences in anther xanthophylls, lutein, neoxanthin, and zeaxanthin, which contribute to the yellow coloration of the petals, reached highly significant levels (Fig. 5I). Furthermore, *AtPOLIB* affects plastid DNA copy numbers in *Arabidopsis thaliana* [35, 36]. To investigate whether *BnaC05.POLIB* performs a similar function in *B. napus*, we examined chloroplast DNA (ctDNA) levels in rapeseed at different growth stages (3rd-leaf and 7th-leaf stages). Real-Time PCR (RT-PCR) analysis revealed that the *cf* mutant exhibited a reduced chloroplast DNA copy number at all time points compared to WT. Specifically, ctDNA levels in the *cf* mutant were reduced by 30% at the 3rd-leaf stage and by 50% at the 7th-leaf stage (Fig. 5J). These results suggest that *BnaC05.POLIB* influences chromoplast development in cells by modulating plastid genome copy number, thereby reducing carotenoid synthesis and accumulation and promoting the formation of chimeric petals in *B. napus*.

## Discussion

### New insights into influencing flower color in *B. napus*, especially chimeric flowers

Most petals of *B. napus* are yellow, with a few varieties exhibiting white (greenish white, creamy white, pure white) and orange-red colors [37]. Recent advances in gene editing and distant hybridization have facilitated the development of novel floral colors, including red and blue [38]. However, chimeric floral materials remain rare, and researches on chimeric flower coloration are limited. Yellow-white chimeric rapeseed flowers were first reported in hybrid sterile plant progeny, proposed to result from locally expressed recessive albino genes coupled with developmental impairments in both plants and floral organs [39]. In this study, EMS mutagenesis yielded stable chimeric yellow-white flower materials that maintained their phenotype across different yellow-flowered genetic backgrounds. Flower color regulation depends on pigment synthesis and metabolism, where key pathway genes determine pigmentation patterns. In rapeseed, yellow coloration primarily arises from carotenoid synthesis and accumulation [40]. The carotenoid degradation enzyme *BnaC3.CCD4* becomes inactivated due to transposon insertion prior to petal yellowing [26]. Mutations in *BnaA08.PDS3*, a carotenoid biosynthesis gene, reduce carotenoid production, resulting in creamy-white petals [37]. In *Brassica juncea*, *BjA02.PC1* and *BjB04.PC2* mediate the esterification of purple xanthophylls, neoxanthin, and anthroxanthin, promoting stable carotenoid storage in plastoglobules [41].

In this study, we identified and cloned the chimeric flower gene *BnaC05.POLIB* from rapeseed (*B. napus*). Notably, the gene operates independently of the carotenoid metabolism pathway. Metabolomic analysis of petals confirmed an intact carotenoid biosynthetic pathway, though with reduced total carotenoid content. Since carotenoid synthesis and accumulation take place within chromoplasts, chromoplast size and number directly influence plant coloration [42]. We determined that mutations in *BnaC05.POLIB* disrupt chromoplast formation in specific cell populations, resulting in the characteristic yellow-white chimeric petal phenotype. These findings reveal a novel regulatory mechanism for flower coloration that directly controls the pigment biosynthesis ‘factory’. Carotenoid production occurs in plastids, and impaired plastid development leads to reduced carotenoid synthesis and accumulation. However, as plastids are essential for normal plant growth and development, their regulation must be precisely balanced to maintain optimal pigment production. Interestingly, brown petals also result from carotenoid accumulation. Through crosses between the *cf* mutant and brown-flowered (BF) material, we obtained F_1_ progeny exhibiting brown-white chimeric petals, further supporting the role of plastid development in color patterning (Fig. S9).

### The *cf* mutation exhibits a pure lethal effect.

Green embryos are observed in certain angiosperms, with chlorophyll persisting until seed maturity. These embryos demonstrate functional photosynthetic capacity [43], indicating chlorophyll’s crucial role in seed development. Embryonic photosynthesis is naturally shade-adapted, as development occurs within light-limited environments created by multilayered pod walls, seed coats, and endosperm. Research has established that mutations disrupting chloroplast function impair embryo development [44–46]. Mutations in distinct chloroplast-related genes cause abnormal development at specific stages, potentially arresting embryos at the globular stage or during the globular-to-cotyledon transition [47, 48]. Alternatively, defects may emerge later at heart-shaped or torpedo stages [49–51]. In some cases, developmental abnormalities only become apparent postgermination, resulting in albino seedlings [52, 53].

In *Arabidopsis*, the *gai* (gibberellin-insensitive) mutation is a well-characterized dominant-negative mutation. A single amino acid change in this gene prevents the degradation of the GAI protein, causing it to continuously inhibit growth, thereby mimicking the complete loss of function in the pathway [54]. Similarly, mutations in the *TP53* tumor suppressor gene in humans often act in a dominant-negative manner: a mutated p53 subunit incorporates into the tetrameric transcription factor complex and inactivates the entire complex, promoting cancer development even in the presence of WT subunits [55]. In contrast, many transcription factors exhibit haploinsufficiency—a loss-of-function scenario in which half the normal gene dosage is insufficient [56]. In such cases, knocking out one allele often produces no observable effect, which stands in sharp contrast to the dominant phenotype observed in our point mutation. Our data demonstrate that the single-nucleotide substitution (G → A) acts through a dominant-negative mechanism, whereas the CRISPR-induced deletion results in a simple loss-of-function mutation. The observation that functional homologs can compensate for the deletion but not the point mutation further supports this distinction. As described previously, self-pollinated progeny of *cf* consistently segregated chimeric and yellow flowers. Compared with the WT, developing seeds of the mutant began to exhibit abnormal white syncytial embryos from 10 days after pollination onward, followed by developmental delays relative to the normal green embryos (Fig. S10A). Statistical analysis indicated ~25% of *cf* seeds exhibited these abnormalities, while WT (7–5) seeds developed normally (Fig. S10B). Genotyping confirmed that albino seeds resulted from homozygous mutations (*bnac05.polIb^D742N^*). Developmental tracking revealed these seeds contained albino or greenish embryos (Fig. S10C), collectively demonstrating that *BnaC05.POLIB* plays an essential role in plant seed development.

### Association of yellow-white chimeric flower color formation with plastids in *B. napus*

**Figure 6:**
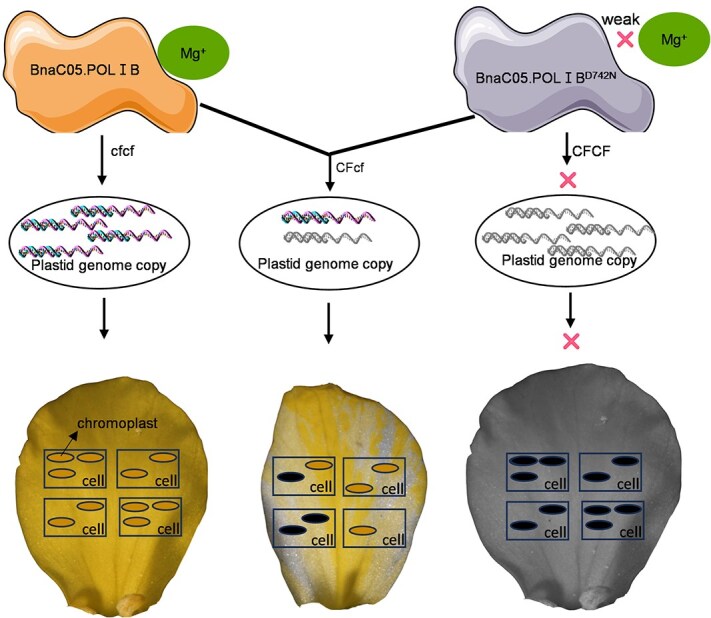
Model for the formation of chimeric flower in *B napus*. A single-nucleotide mutation in BnaC05.POLIB, which disrupts the encoded protein's Mg^2+^-binding capacity (as evidenced by green electron density maps), reduces plastid genome copy number, thereby decreasing plastid-derived chromoplasts per petal cell and ultimately generating a yellow-white chimeric petal phenotype, while homozygous mutants exhibit severely impaired plastid development that leads to lethality.

The stability of the plastid genome is crucial for plastid function. Mutations affecting plastid genome stability can disrupt endoreduplication and the cell cycle, as demonstrated in mutant *reca1/why1/why3* [57]. *Arabidopsis thaliana* encodes two plastid DNA polymerase genes, *POLIA* and *POLIB*, which localize to both mitochondria and chloroplasts and exhibit tissue-specific expression patterns [35]. *polIb* display stunted growth, impaired cell expansion, and additional pleiotropic phenotypes [36]. Here, we cloned the plastid-targeted DNA polymerase *BnaC05.POLIB* from *B. napus*. A missense mutation at amino acid position D742, which coordinates Mg^2+^ ions critical for the bimetallic catalytic mechanism of DNA polymerases [58], was identified. D742 coordinates one of the two Mg^2+^ ions required for catalysis, facilitating dNTP positioning and stabilizing the transition state during nucleophilic attack on the dNTP α-phosphate group. Substitution of D742 with asparagine (N742) severely impairs polymerase activity, likely increasing DNA replication errors due to active-site destabilization and nucleotide misincorporation, thereby elevating mutagenesis rates. In heterozygotes, reduced POLIB activity decreases plastid genome copy number, impairing chromoplast development in select petal cells. This defect leads to diminished carotenoid synthesis and accumulation, ultimately causing flower color chimerism (Fig. 6).

## Materials and methods

### Plant materials and planting management

The *cf* mutant, exhibiting yellow-white chimeric flowers in *B. napus*, was obtained through EMS mutagenesis of the 7–5 accession, a yellow-flowered WT. Xiaoyun, a model *B. napus* accession employed in functional genomics studies [59]. was crossed with *cf* to generate F_1_ hybrids. F_2_ populations were generated by self-pollinating the F_1_ hybrids. Initial genetic mapping was conducted using a total of 704 M_5_ generation individuals, while fine mapping employed an additional 4600 individuals from the F_2_ population (derived from the *cf* × Xiaoyun cross). Both F₂ mapping populations and transgenic rapeseed lines were grown in a speed breeding greenhouse under predefined conditions: 22°C–24°C temperatures, 22-h light/2-h dark photoperiod, and 650 μmol·m^−2^·s^−1^ PPFD measured 10 cm below light sources. [60]. Additionally, the M_5_ population was grown at the experimental field of Huazhong Agricultural University in Wuhan, China.

### Analysis of carotenoids

Carotenoid content was quantified using spectrophotometric methods following established protocols [61]. Fresh petals from the *cf* mutant and the 7–5 WT were lyophilized. The resulting freeze-dried tissue was cryogenically ground in a mixer mill (1.5 min, 30 Hz) to obtain a fine powder, which was subsequently stored at −80°C. Fifty milligrams of the powdered sample was vortexed with a mixed solvent system comprising 0.5 ml of hexane, acetone, and ethanol (1:1:1, v/v/v) for 20 min at ambient temperature to ensure sufficient extraction. Subsequently, the extracts were subjected to analysis using UPLC-APCI-MS/MS system [62]. During the identification of carotenoids, accurate identification of the target compounds was accomplished by comparing the UV–Vis spectral characteristics and chromatographic retention times of the samples with those of reference standards and literature data. Absolute carotenoid content was determined by MetWare (http://www.metware.cn/) using an AB Sciex QTRAP 6500 LC–MS/MS platform.

### Transmission electron microscopy

Petals harvested from the *cf* mutant and the 7–5 WT plants were subjected to ultrastructural analysis via transmission electron microscopy (TEM) according to established protocols [63] . Petal tissue was sectioned into 1-mm^3^ blocks and fixed in 2.5% (w/v) glutaraldehyde dissolved in 0.1 mol/l phosphate-buffered saline (PBS; pH 7.2) at 4°C overnight. Fixed specimens were rinsed three times with PBS buffer at room temperature (30 min per rinse, minimum), followed by postfixation in 1% osmium tetroxide solution for 2 h. This was followed by dehydration through a graded acetone series and subsequent resin infiltration. Following 48 h of polymerization, ultrathin sections were mounted on grids, stained with uranyl acetate, and visualized using a Hitachi H-7650 TEM at an accelerating voltage of 80 kV. We choose three biological replicates to analyze per genotype, with each replicate comprising at least three independently examined ultrathin sections. Chromoplast and plasmodesmata quantification in petal cells was performed by examining 15 cells and 15 chromoplasts per replicate across two independent biological replicates.

### MutMap analysis

Thirty petals with yellow and chimeric coloration were selected in the progeny of M_5_. Genomic DNA was extracted via the CTAB method, followed by library construction and sequencing on the Illumina NovaSeq 6000 platform (Illumina, San Diego, CA, USA). Clean reads were filtered using fastp, and the filtered reads were aligned to the *B. napus* reference genome ZS11 (http://cbi.hzau.edu.cn/cgi-bin/rape/download_ext) via bwa-mem2. SNPs and insertions/deletions (InDels) were identified and filtered using the Genome Analysis Toolkit (GATK), with heterozygous and deletion variants excluded from the analysis. Δ(SNP-index) was calculated as described by Takagi [64]. Finally, QTL-seq analysis was conducted using the R package easyQTLseq (https://github.com/laowang1992/easyQTLseq.git).

### Marker development and genetic mapping

Following quality control, the data were filtered and aligned to the Xiaoyun reference genome. Based on resequencing data, we developed InDel and SNP markers to map the CF locus. (Table S6) [59]. InDel detection was performed using 8% polyacrylamide denaturing gels and visualized via silver staining, whereas SNP identification was conducted through direct sequencing of PCR amplicons. All amplifications used a standard protocol: initial denaturation (95°C, 3 min); 35 cycles of 95°C (15 s), 58°C (15 s), and 72°C (30 s); concluding with final extension (72°C, 5 min). Each reaction mixture contained 2× Taq Master Mix (Vazyme, P111–01-AA).

### Vector construction and transgenic experiments

The construction of complementary vectors was initiated with the amplification of *Bna.C05POLIB* containing the mutation site was amplified from the *cf* mutant with unique primers (Table S6), along with its own 1592-bp promoter and 966-bp 3′ untranslated region. The obtained fragment was then ligated into the pFGC5941 vector using a homologous recombination enzyme (Vazyme, C112-01). For CRISPR/Cas9 vector assembly, the *BnaC05.POLIB* sequence was analyzed using Geneious Prime software (v2023.2) to identify two 20-nt gRNA target sites adjacent to the mutation site, each containing an NGG protospacer adjacent motif (PAM). These gRNAs were cloned into the pKSE401 vector backbone using established protocols [65]. All constructs were transformed into Xiaoyun using the method described by previous researchers, utilizing rapeseed hypocotyls through GV3101 Agrobacterium. Mutation sites were PCR-amplified with specific primers, and amplicons were subjected to Illumina-based next-generation sequencing to detect CRISPR/Cas9-induced mutations in T0 transgenic lines and evaluate complementation efficiency. All positive monocots were analyzed by sequence comparison to confirm the type of mutation.

### Chloroplast genome copy number assay

Chloroplast genome copy number was quantified via Absolute Quantification PCR (AQ-PCR) following established methods [36]. First, a TA-clone plasmid of the gene to be quantified (from the chloroplast genome) was constructed, and its concentration was determined using NanoDrop (Table S5). Six gradient dilutions (10×) of standards were prepared, and both standards and DNA templates (from 3rd- and 7th-leaf stage plants) underwent RT-PCR amplification to determine Ct values. A standard curve plotting known concentrations against observed Ct values enabled calculation of test sample copy numbers by interpolating their Ct values. Expression levels of plastid genes were normalized against single-copy nuclear reference genes (BnaC09G0363100ZS).

### RNA-seq analysis

Total RNA was extracted from petals at full bloom. RNA sequencing (including library construction and sequencing) was conducted by Shanghai Paison Company. Quality control and alignment of RNA-seq data were performed using the same methods as those employed in BSA-seq. Transcript abundance was quantified using featureCounts [66], and gene expression levels were normalized using the trimmed mean of M-values (TMM) method. Functional enrichment analysis was carried out using the clusterProfiler R package.

## Supplementary Material

Web_Material_uhaf276
