# Supplementary material for: A single nucleotide mutation of BnaC05.POLIB creates yellow-white chimeric flower in Brassica napus
Source: Hortic Res. 2026 Jan 1;13(1):uhaf276. doi: 10.1093/hr/uhaf276 (PMC12903450; doi:10.1093/hr/uhaf276)
Supplement: Web_Material_uhaf276 [file web_material_uhaf276.zip › Supplementary figure1-10.docx]

**Supplementary figure**


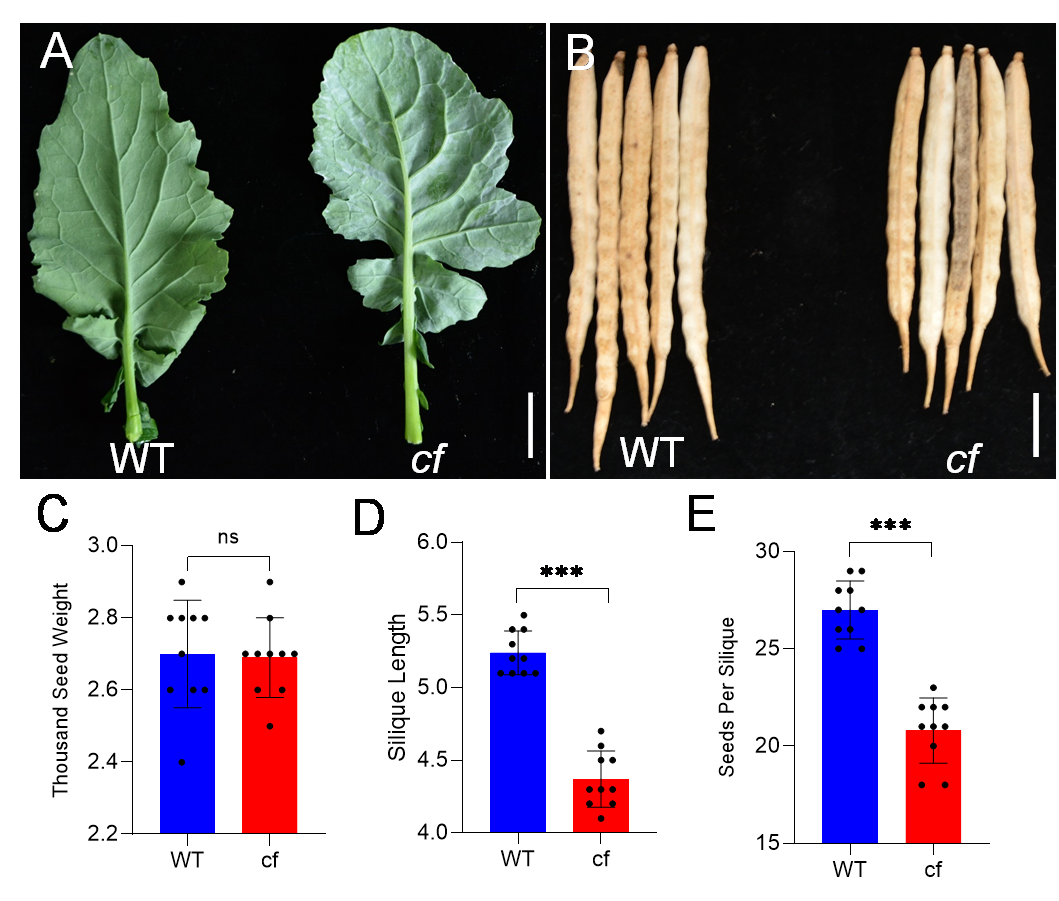


Figure S1. Phenotype and yield-related traits between two parents. (A) Comparison of Leaf Phenotypes. WT (left), *cf*(right), Bar, 1cm. (B) Comparison of silique between two parents. WT (left), *cf*(right), Bar, 1cm. (C-E) Statistical comparison of yield-related traits. n=10 (c) Thousand-seed weight (TSW), (D) silique length, (E) Seeds per silique


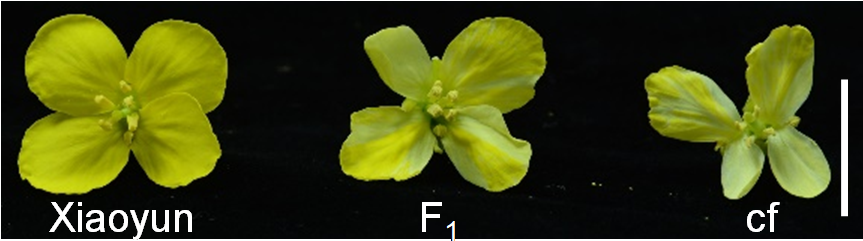


Figure S2. Petal phenotypes of XiaoYun(left), *cf*(right)and their F_1_ (middle)hybrid progeny. Bar, 1cm


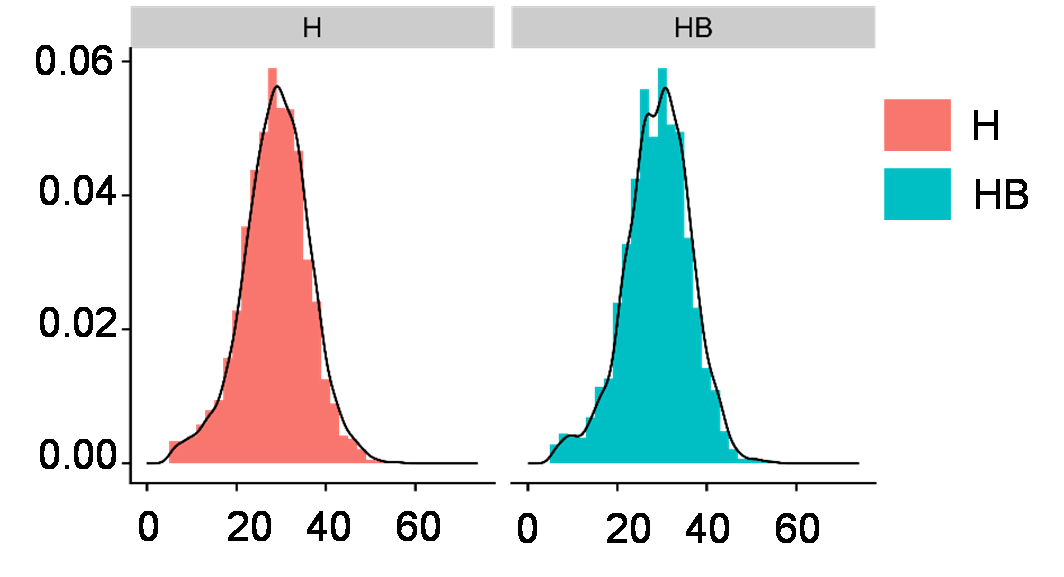


Figure S3. Sequencing depth of both pools. The horizontal coordinate represents the sequencing depth and the vertical coordinate represents the sequencing density. The figure shows that the sequencing depth of both pools is concentrated at 30 X. H, WT pool, HB, mutant pool.


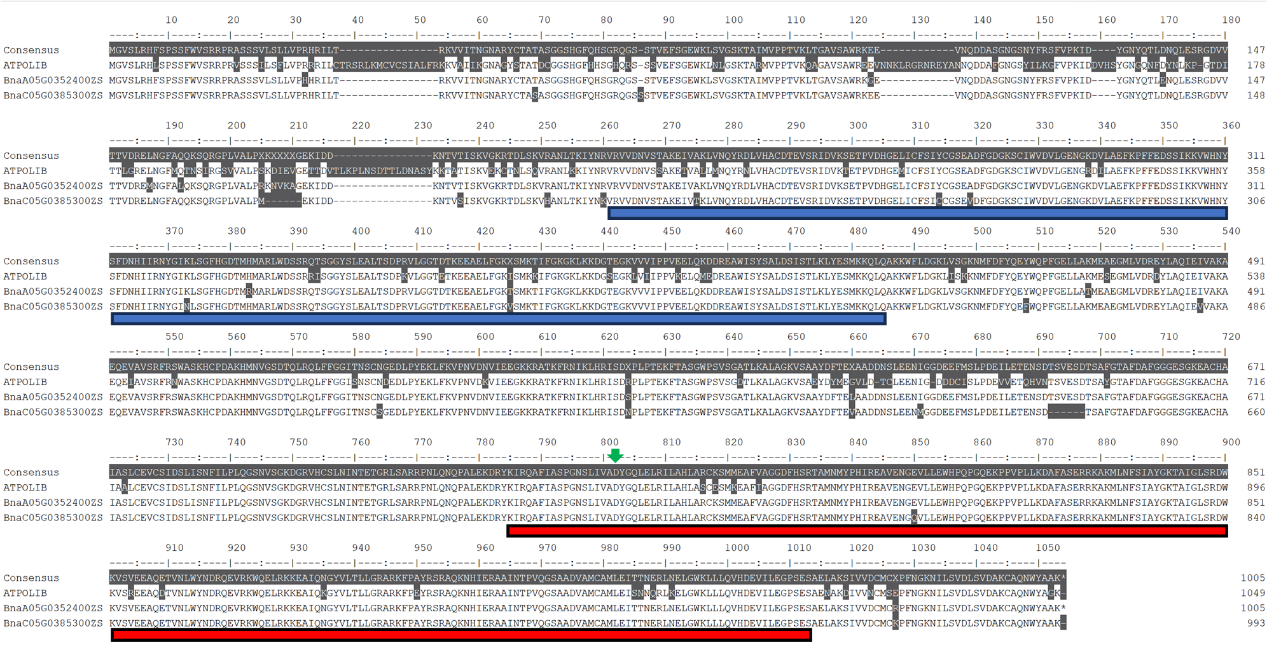


Figure S4 Comparative analysis of homologous gene sequences. The blue box indicates the predicted 35EXOc domain, the red box indicates the predicted POLAc domain, and the green arrow marks the mutated site.


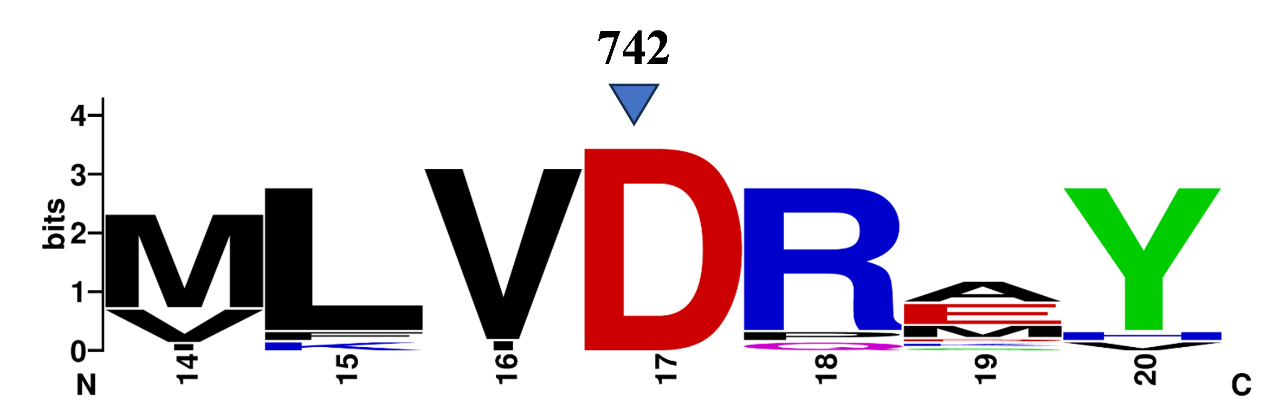


Figure S5. Conservation analysis of mutation sites in *BnaC05.POLⅠB*. Different colors represent different amino acids, and the size of each letter correlates with the degree of conservation. The number 742 indicates the positions of amino acid mutations in BnaC05.POLⅠB protein.


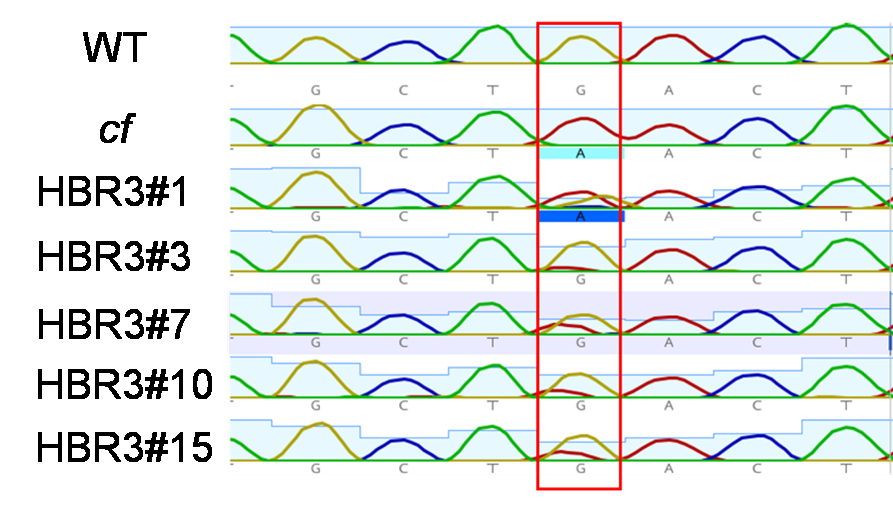


Figure S6. Identification of complementary transgenic plants positive by sequencing. The left side represents the transgenic lines and the red box on the right side indicates the presence of the mutant site, residue G represent WT, residue A represent G, Twin peaks represent positive transgenic plants.


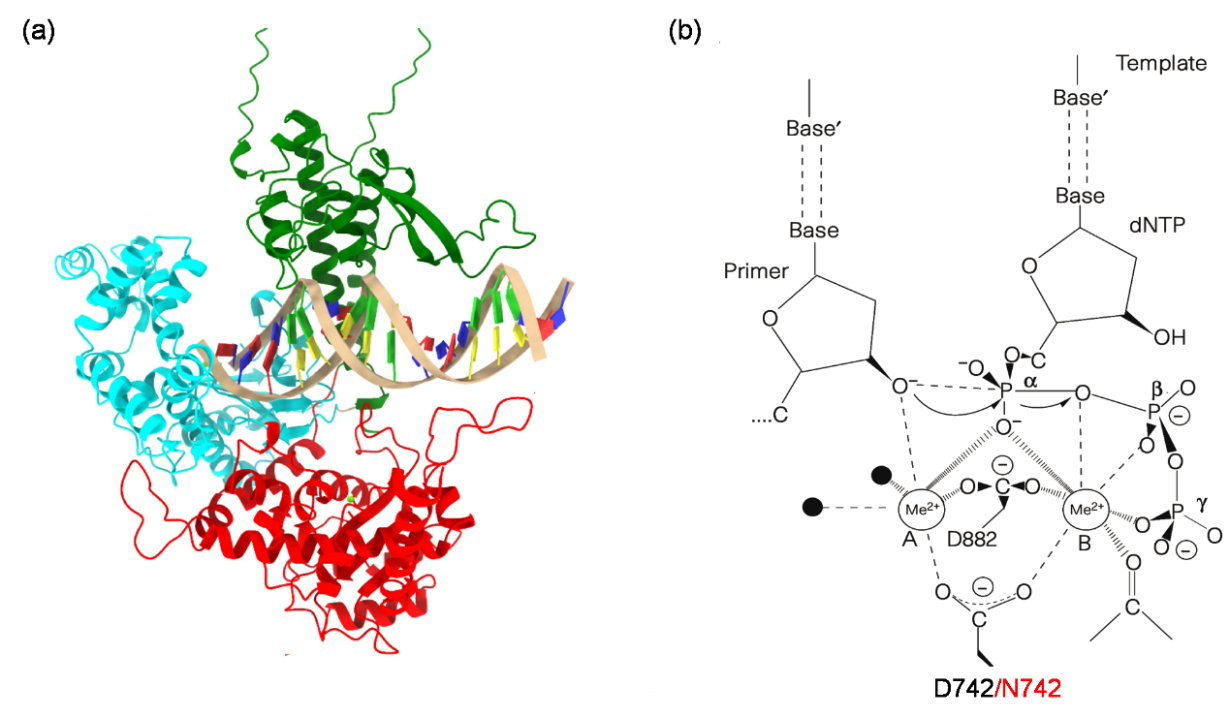


Figure S7. Overall structure of the BnaC05-POLⅠB DNA complex.

(a) The complex adopts a classic polymerase architecture, with the thumb, palm, and fingers domains colored in green, red, and cyan, respectively. The DNA template and primer strands are shown in orange.

(b) Schematic representation of the two-metal-ion mechanism of DNA polymerase catalysis. The wild-type enzyme (left) utilizes two divalent metal ions (Mg2+) to coordinate the incoming dNTP and facilitate the nucleophilic attack on the α-phosphate. The D742N mutant enzyme （color red） is unable to efficiently coordinate one of the metal ions, leading to a reduction in catalytic activity. The template and primer strands, the incoming dNTP, and the catalytic metal ions are shown the critical aspartate residue (D742) and its mutant counterpart (N742) are highlighted.


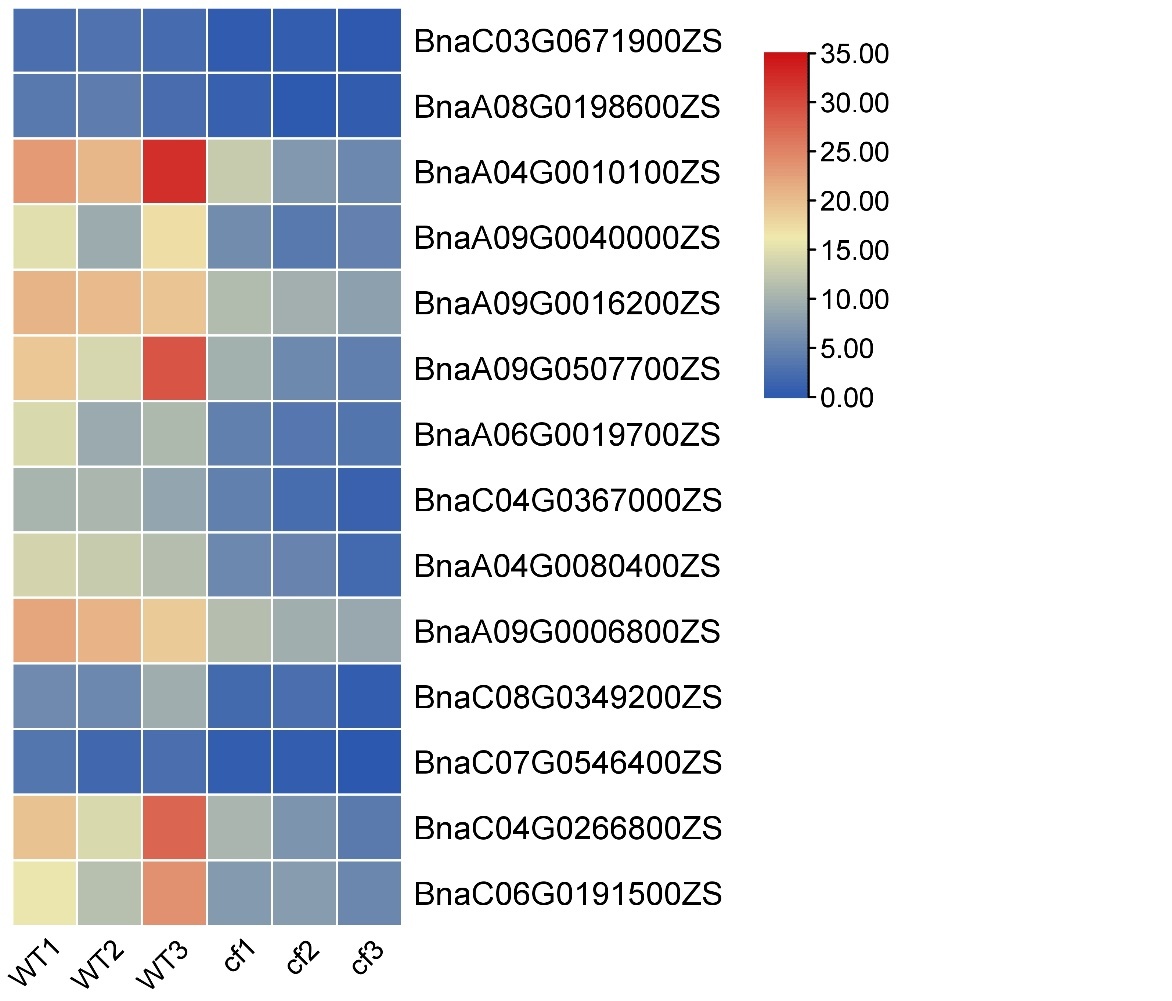


Figure S8. Comparative analysis of heatmaps for genes associated with plastid development in petals.


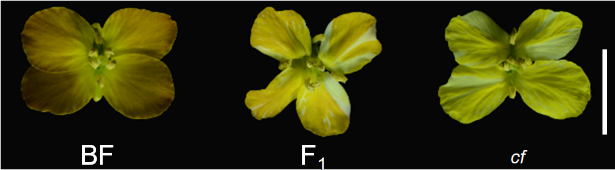


Figure S9. Petal color phenotypes in *cf,* BF (brown flower) and their F_1_ progeny. Bar, 1cm.


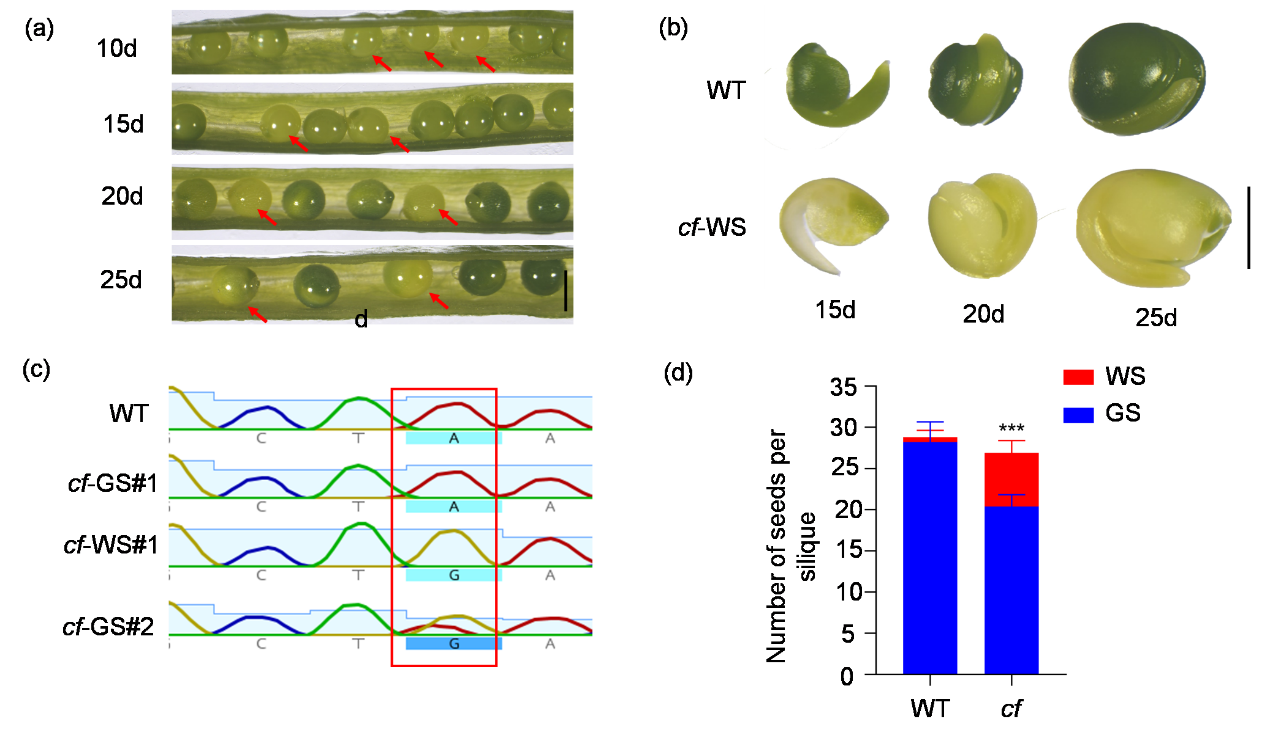

Figure S10 *BnaC05.POLIB* affects the development of *B napus* seeds.

(a) Observations of seed development in self-pollinated angiosperms of the *cf* mutant 10 days-25 days after flowering. Red arrows indicate problematic seeds. Bar,1mm.

(b) Comparison between *cf* mutant white embryos and 7-5 green embryos (15-25DAY) Bar, 1mm.

(c) Seeds genotyped in both states of the *cf* mutant, *cf*-GS indicates green seeds in *cf* and *cf*-WS indicates white seeds. *cf*-GS#1 indicates a genotype consistent with WT, which exhibits green color, and *cf*-GS#2 indicates a heterozygous genotype in the seed that carries the mutant locus and also exhibits green color.

(d) Statistics on the number of problematic seeds in 20-days siliques between WT and *cf*, red bars indicate abnormal white seeds, blue bars indicate normal green seeds, n=10. ****P*<0.001.
