# Supplementary material for: A single nucleotide mutation of BnaC05.POLIB creates yellow-white chimeric flower in Brassica napus
Source: Hortic Res. 2026 Jan 1;13(1):uhaf276. doi: 10.1093/hr/uhaf276 (PMC12903450; doi:10.1093/hr/uhaf276)
Supplement: Web_Material_uhaf276 [file web_material_uhaf276.zip › Supplementary Figure S4.pdf]

```
                  10        20        30        40        50        60        70        80        90       100       110       120       130       140       150       160       170       180
Consensus         MGVSLRHFSPSSFWVSRRPRASSSVLSLLVPRHRILT---------------RKVVITNGNARYCTATASGGSHGFQHSGRQGS-STVEFSGEWKLSVGSKTAIMVPPTVKLTGAVSAWRKEE-----------VNQDDASGNGSNYFRSFVPKID----YGNYQTLDNQLESRGDVV   147
ATPOLIB           MGVSLRHLSPSSFWVSRRPRVSSSILSFLVPRRRILCTRSRLKMCVCSIALFRKKVAIIKGNAGYSTATDCGGSHGFHHSGHQRS-SSVEFSGEWKLNLGSKTARMVPPTVKQAGAVSAWREEVNNKLRGRNREYANNQDDAFGNGSYILKGFVPKIDDVHSYGNGQNFDYNLKP-GTDI   178
BnaA05G0352400ZS  MGVSLRHFSPSSFWVSRRPRASSSVLSLLVPHHRILT---------------RKVVITNGNARYCTATASGGSHGFQHSGRQGS-STVEFSGEWKLSVGSKTAIMVPPTVKLTGAVSAWRKGE-----------VNQDDASGNGSNYFRSFVPKID----YGNYQTLENQLESRGDVV   147
BnaC05G0385300ZS  MGVSLRHFSPSSFWVSRRPRASSSVLSLLVPRHRILT---------------RKVVITNGNARYCTASASGGSHGFQHSGRQGSSSTVEFSGEWKLSVGSKTAIMVPPTVKLTGAVSAWRKEE-----------VNQDDASGNGSNYFRSFVPKID----YGNYQTLDNQLESRGDVV   148

                 190       200       210       220       230       240       250       260       270       280       290       300       310       320       330       340       350       360
Consensus         TTVDRELNGFAQQKSQRGPLVALPXKXXXXGEKIDD---------------KNTVTISKVGKRTDLSKVRANLTKIYNRVRVVDNVSTAKEIVAKLVNQYRDLVHACDTEVSRIDVKSETPVDHGELICFSIYCGSEADFGDGKSCIWVDVLGENGKDVLAEFKPFFEDSSIKKVWHNY   311
ATPOLIB           TTLGRELNGFMQTNSIRGSVVALPSKDIEVGETTDVTLKPLNSDTTLDNASYKKTATISKVEKCTNLSQVRANLKKIYNRVRVVDNVSSAKETVALLMNQYRNLVHACDTEVSRIDVKTETPVDHGEMICFSIYCGSEADFGDGKSCIWVDVLGENGRDILAEFKPFFEDSSIKKVWHNY   358
BnaA05G0352400ZS  TTVDREMNGFALQKSQRGPLVALPRKNVKAGEKIDD---------------KNTVTISKVGKRTDLSKVRANLTKIYNRVRVVDNVSTAKEIVAKLVNQYRDLVHACDTEVSRIDVKSETPVDHGELICFSIYCGSEADFGDGKSCIWVDVLGENGKDVLAEFKPFFEDSSIKKVWHNY   311
BnaC05G0385300ZS  TTVDRELNGFAQQKSQRGPLVALPM------EKIDD---------------KNTVSISKVGKRTDLSKVHANLTKIYNKVRVVDNVSTAKEIVTKLVNQYRDLVHACDTEVSRIDVKSETPVDHGELICFSICCGSEVDFGDGKSCIWVDVLGENGKDVLAEFKPFFEDSSIKKVWHNY   306

                 370       380       390       400       410       420       430       440       450       460       470       480       490       500       510       520       530       540
Consensus         SFDNHIIRNYGIKLSGFHGDTMHMARLWDSSRQTSGGYSLEALTSDPRVLGGTDTKEEAELFGKXSMKTIFGKGKLKKDGTEGKVVVIPPVEELQKDDREAWISYSALDSISTLKLYESMKKQLQAKKWFLDGKLVSGKNMFDFYQEYWQPFGELLAKMEAEGMLVDREYLAQIEIVAKA   491
ATPOLIB           SFDNHIIRNYGIKLSGFHGDTMHMARLWDSSRRISGGYSLEALTSDPKVLGGTETKEEAELFGKISMKKIFGKGKLKKDGSEGKLVIIPPVKELQMEDREAWISYSALDSISTLKLYESMKKQLQAKKWFLDGKLISKKNMFDFYQEYWQPFGELLAKMESEGMLVDRDYLAQIEIVAKA   538
BnaA05G0352400ZS  SFDNHIIRNYGIKLSGFHGDTMRMARLWDSSRQTSGGYSLEALTSDPRVLGGTDTKEEAELFGKTSMKTIFGKGKLKKDGTEGKVVVIPPVEELQKDDREAWISYSALDSISTLKLYESMKKQLQAKKWFLDGKLVSGKNMFDFYQEYWQPFGELLATMEAEGMLVDREYLAQIEIVAKA   491
BnaC05G0385300ZS  SFDNHIIRNYGINLSGFHGDTMHMARLWDSSRQTSGGYSLEALTSDPRVLGGTDTKEEAELFGKVSMKTIFGKGKLKKDGTEGKVVVIPPVEELQKDDREAWISYSALDSISTLKLYESMKKQLQAKKWFLDGKLVSGKNMFDFYQEFWQPFGELLAKMEAEGMLVDREYLAQIEVVAKA   486

                 550       560       570       580       590       600       610       620       630       640       650       660       670       680       690       700       710       720
Consensus         EQEVAVSRFRSWASKHCPDAKHMNVGSDTQLRQLFFGGITNSCNGEDLPYEKLFKVPNVDNVIEEGKKRATKFRNIKLHRISDXPLPTEKFTASGWPSVSGATLKALAGKVSAAYDFTEXAADDNSLEENIGGDEEFMSLPDEILETENSDTSVESDTSAFGTAFDAFGGGESGKEACHA   671
ATPOLIB           EQEIAVSRFRNWASKHCPDAKHMNVGSDTQLRQLFFGGISNSCNDEDLPYEKLFKVPNVDKVIEEGKKRATKFRNIKLHRISDRPLPTEKFTASGWPSVSGDTLKALAGKVSAEYDYMEGVLD-TCLEENIG-DDDCISLPDEVVETQHVNTSVESDTSAYGTAFDAFGGGESGKEACHA   716
BnaA05G0352400ZS  EQEVAVSRFRSWASKHCPDAKHMNVGSDTQLRQLFFGGITNSCNGEDLPYEKLFKVPNVDNVIEEGKKRATKFRNIKLHRISDSPLPTEKFTASGWPSVSGATLKALAGKVSAAYDFTELAADDNSLEENIGGDEEFMSLPDEILETENSDTSVESDTSAFGTAFDAFGGGESGKEACHA   671
BnaC05G0385300ZS  EQEVAVSRFRSWASKHCPDAKHMNVGSDTQLRQLFFGGITNSCSGEDLPYEKLFKVPNVDNVIEEGKKRATKFRNIKLHRISDNPLPTEKFTASGWPSVSGATLKALAGKVSAAYDFTEVAADDNSLEENMGGDEEFMSLPDEILETENSD------TSAFGTAFDAFGGGESGKEACHA   660

                 730       740       750       760       770       780       790       800       810       820       830       840       850       860       870       880       890       900
Consensus         IASLCEVCSIDSLISNFILPLQGSNVSGKDGRVHCSLNINTETGRLSARRPNLQNQPALEKDRYKIRQAFIASPGNSLIVADYGQLELRILAHLARCKSMMEAFVAGGDFHSRTAMNMYPHIREAVENGEVLLEWHPQPGQEKPPVPLLKDAFASERRKAKMLNFSIAYGKTAIGLSRDW   851
ATPOLIB           IAALCEVCSIDSLISNFILPLQGSNVSGKDGRVHCSLNINTETGRLSARRPNLQNQPALEKDRYKIRQAFIASPGNSLIVADYGQLELRILAHLASCESMKEAFIAGGDFHSRTAMNMYPHIREAVENGEVLLEWHPQPGQEKPPVPLLKDAFASERRKAKMLNFSIAYGKTAIGLSRDW   896
BnaA05G0352400ZS  IASLCEVCSIDSLISNFILPLQGSNVSGKDGRVHCSLNINTETGRLSARRPNLQNQPALEKDRYKIRQAFIASPGNSLIVADYGQLELRILAHLARCKSMMEAFVAGGDFHSRTAMNMYPHIREAVENGEVLLEWHPQPGQEKPPVPLLKDAFASERRKAKMLNFSIAYGKTAIGLSRDW   851
BnaC05G0385300ZS  IASLCEVCSIDSLISNFILPLQGSNVSGKDGRVHCSLNINTETGRLSARRPNLQNQPALEKDRYKIRQAFIASPGNSLIVADYGQLELRILAHLARCKSMMEAFVAGGDFHSRTAMNMYPHIREAVENGQVLLEWHPQPGQEKPPVPLLKDAFASERRKAKMLNFSIAYGKTAIGLSRDW   840

                 910       920       930       940       950       960       970       980       990      1000      1010      1020      1030      1040      1050
Consensus         KVSVEEAQETVNLWYNDRQEVRKWQELRKKEAIQNGYVLTLLGRARKFPAYRSRAQKNHIERAAINTPVQGSAADVAMCAMLEITTNERLNELGWKLLLQVHDEVILEGPSESAELAKSIVVDCMCXPFNGKNILSVDLSVDAKCAQNWYAAK*                             1005
ATPOLIB           KVSREEAQDTVNLWYNDRQEVRKWQELRKKEAIQKGYVLTLLGRARKFPEYRSRAQKNHIERAAINTPVQGSAADVAMCAMLEISNNQRLKELGWKLLLQVHDEVILEGPSESAENAKDIVVNCMSEPFNGKNILSVDLSVDAKCAQNWYAGK-                             1049
BnaA05G0352400ZS  KVSVEEAQETVNLWYNDRQEVRKWQELRKKEAIQNGYVLTLLGRARKFPAYRSRAQKNHIERAAINTPVQGSAADVAMCAMLEITTNERLNELGWKLLLQVHDEVILEGPSESAELAKSIVVDCMCRPFNGKNILSVDLSVDAKCAQNWYAAK*                             1005
BnaC05G0385300ZS  KVSVEEAQETVNLWYNDRQEVRKWQELRKKEAIQNGYVLTLLGRARKFPAYRSRAQKNHIERAAINTPVQGSAADVAMCAMLEITTNERLNELGWKLLLQVHDEVILEGPSESAELAKSIVVDCMCKPFNGKNILSVDLSVDAKCAQNWYAAK-                              993
```
