# Supplementary material for: A single nucleotide mutation of BnaC05.POLIB creates yellow-white chimeric flower in Brassica napus
Source: Hortic Res. 2026 Jan 1;13(1):uhaf276. doi: 10.1093/hr/uhaf276 (PMC12903450; doi:10.1093/hr/uhaf276)
Supplement: Web_Material_uhaf276 [file web_material_uhaf276.zip › Supplementary Table1-6.docx]

Table S1. Quality control of MutMap resequencing data for mixed pool samples of the petal M_5_ population

| Sample | H | HB |
| --- | --- | --- |
| total_reads.raw | 268532336 | 269471908 |
| total_bases.raw | 40484556604 | 40635070316 |
| q20_bases.raw | 38880652337 | 39300551131 |
| q30_bases.raw | 36404731558 | 37122529636 |
| q20_rate.raw | 0.960382 | 0.967158 |
| q30_rate.raw | 0.899225 | 0.913559 |
| gc_content.raw | 0.378789 | 0.379142 |
| total_reads.clean | 265676746 | 267106602 |
| total_bases.clean | 39760163740 | 39992789032 |
| q20_bases.clean | 38350438376 | 38834156410 |
| q30_bases.clean | 35970240261 | 36737746383 |
| q20_rate.clean | 0.964544 | 0.971029 |
| q30_rate.clean | 0.90468 | 0.918609 |
| gc_content.clean | 0.377909 | 0.378363 |
| effective.rate | 0.982106933 | 0.984193917 |

| Gene ID | Position | Arabidopsis homologue | | | Gene annotation |
| --- | --- | --- | --- | --- | --- |
| BnaC05G0384300ZS | [43816741..43818714 (-)](http://cbi.hzau.edu.cn/cgi-bin/bnapus/gb2/gbrowse/ZS11v0?name=BnaC05G0384300ZS) | | AT3G20600 | Protein NDR1 OS=Arabidopsis thaliana GN=NDR1 PE=1 SV=1 | |
| BnaC05G0384400ZS | [43818715..43819111 (-)](http://cbi.hzau.edu.cn/cgi-bin/bnapus/gb2/gbrowse/ZS11v0?name=BnaC05G0384400ZS) | | AT3G20600 | Protein NDR1 OS=Arabidopsis thaliana GN=NDR1 PE=1 SV=1 | |
| BnaC05G0384500ZS | [43827825..43828682 (+)](http://cbi.hzau.edu.cn/cgi-bin/bnapus/gb2/gbrowse/ZS11v0?name=BnaC05G0384500ZS) | | AT3G20580 | COBRA-like protein 10 OS=Arabidopsis thaliana GN=COBL10 PE=2 SV=1 | |
| BnaC05G0384600ZS | 43828715..43829044 (+) | | AT3G20580 | COBRA-like protein 10 OS=Arabidopsis thaliana GN=COBL10 PE=2 SV=1 | |
| BnaC05G0384700ZS | [43832207..43832920 (+)](http://cbi.hzau.edu.cn/cgi-bin/bnapus/gb2/gbrowse/ZS11v0?name=BnaC05G0384700ZS) | | AT3G20570 | Early nodulin-like protein 1 OS=Arabidopsis thaliana GN=At2g25060 PE=1 SV=2 | |
| BnaC05G0384800ZS | [43833194..43837208 (-)](http://cbi.hzau.edu.cn/cgi-bin/bnapus/gb2/gbrowse/ZS11v0?name=BnaC05G0384800ZS) | | AT3G20560 | Protein disulfide-isomerase 5-3 OS=Arabidopsis thaliana GN=PDIL5-3 PE=2 SV=1 | |
| BnaC05G0384900ZS | [43839662..43840300 (-)](http://cbi.hzau.edu.cn/cgi-bin/bnapus/gb2/gbrowse/ZS11v0?name=BnaC05G0384900ZS) | | AT3G20557 | VASCULAR-RELATED UNKNOWN PROTEIN 3, VUP3 | |
| BnaC05G0385000ZS | [43841747..43841973 (+)](http://cbi.hzau.edu.cn/cgi-bin/bnapus/gb2/gbrowse/ZS11v0?name=BnaC05G0385000ZS) | | AT1G52590 | DCC family protein At1g52590, chloroplastic OS=Arabidopsis thaliana GN=At1g52590 PE=2 SV=1 | |
| BnaC05G0385100ZS | [43844163..43844430 (+)](http://cbi.hzau.edu.cn/cgi-bin/bnapus/gb2/gbrowse/ZS11v0?name=BnaC05G0385100ZS) | | AT5G03300 | Adenosine kinase 2 OS=Arabidopsis thaliana GN=ADK2 PE=1 SV=1 | |
| BnaC05G0385200ZS | [43845903..43846533 (-)](http://cbi.hzau.edu.cn/cgi-bin/bnapus/gb2/gbrowse/ZS11v0?name=BnaC05G0385200ZS) | | AT1G50900 | Protein LHCP TRANSLOCATION DEFECT OS=Arabidopsis thaliana GN=LTD PE=1 SV=1 | |
| BnaC05G0385300ZS | [43847220..43851754 (-)](http://cbi.hzau.edu.cn/cgi-bin/bnapus/gb2/gbrowse/ZS11v0?name=BnaC05G0385300ZS) | | AT3G20540 | DNA polymerase I B, chloroplastic/mitochondrial OS=Arabidopsis thaliana GN=POLIB PE=2 SV=1 | |
| BnaC05G0385400ZS | [43853480..43858439 (-)](http://cbi.hzau.edu.cn/cgi-bin/bnapus/gb2/gbrowse/ZS11v0?name=BnaC05G0385400ZS) | | AT3G20520 | Glycerophosphodiester phosphodiesterase GDPDL5 OS=Arabidopsis thaliana GN=GDPDL5 PE=2 SV=1 | |
| BnaC05G0385500ZS | [43859116..43860235 (-)](http://cbi.hzau.edu.cn/cgi-bin/bnapus/gb2/gbrowse/ZS11v0?name=BnaC05G0385500ZS) | | AT3G20510 | Protein FATTY ACID EXPORT 6 OS=Arabidopsis thaliana GN=FAX6 PE=3 SV=1 | |
| BnaC05G0385600ZS | [43863569..43866425 (-)](http://cbi.hzau.edu.cn/cgi-bin/bnapus/gb2/gbrowse/ZS11v0?name=BnaC05G0385600ZS) | | AT3G20500 | Purple acid phosphatase 18 OS=Arabidopsis thaliana GN=PAP18 PE=2 SV=1 | |

Table S2. Chimeric floral color locus candidate genes analysed in a 52kb region in reference genome of *B. napu*s ZS11

Table S3. Analysis of protein sequence identity between *BnaC05.POLIB* and *ATPOLIB*

| Gene | Identity (%) | | | | | |
| --- | --- | --- | --- | --- | --- | --- |
|  | *ATPOLIA* | *ATPOLIB* | *BnaC05.POLIB* | *BnaA05.POLIB* | *BraPOLIB* | *BolPOLIB* |
| *ATPOLIA* | 100 | 79.89 | 78.58 | 78.9 | 55.01 | 78.33 |
| *ATPOLIB* | 79.89 | 100 | 86.24 | 87 | 60.37 | 86.07 |
| *BnaC05.POLIB* | 78.58 | 86.24 | 100 | 97.42 | 66.62 | 98.99 |
| *BnaA05.POLIB* | 78.9 | 87 | 97.42 | 100 | 68.3 | 97.61 |
| *BraPOLIB* | 55.01 | 60.37 | 66.62 | 68.3 | 100 | 66.69 |
| *BolPOLIB* | 78.33 | 86.07 | 98.99 | 97.61 | 66.69 | 100 |

Table S4. Genetic analysis of transgenic complementary plants of T_1_ generation

| Transgenic lines | Totals | Yellow flowers | Chimeric flower | Expected ratio | *Χ^2^* | *P-value* |
| --- | --- | --- | --- | --- | --- | --- |
| HBR3#1 | 48 | 11 | 36 | 1：3 | 0.08 | >0.05 |
| HBR3#3 | 48 | 13 | 35 | 1：3 | 0.11 | >0.05 |
| HBR3#7 | 48 | 0 | 48 | 1：3 | 16 | <0.05 |
| HBR3#10 | 48 | 10 | 38 | 1：3 | 0.44 | >0.05 |
| HBR3#15 | 48 | 0 | 48 | 1：3 | 16 | <0.05 |

Table S5. Carotenoid components in yellow and yellow-white flower petals of *Brassica napus*

| **Compounds** | **Yellow petal（μg/g）** | **Yellow and white petal(μg/g)** |
| --- | --- | --- |
| *α*-carotene | 0.65±0.07 | 0.83±0.06 |
| β-carotene | 3.06±0.18 | 2.1±0.19 |
| (E/Z)-phytoene | 16.05±2.46 | 29.74±11.63 |
| γ-carotene | 0.04±0.06 | 0.07±0.05 |
| phytofluene | 3.91±1.15 | 7.39±2.64 |
| capsanthin | 0.06±0.04 | 0.1±0.01 |
| antheraxanthin | 21.54±1.01*** | 7.52±1.86 |
| echinenone | 0±0 | 0±0 |
| *α*-cryptoxanthin | 9.27±0.77 | 7.92±1.95 |
| β-cryptoxanthin | 2.53±0.12 | 1.52±0.29 |
| Lutein | 292.81±14.07*** | 155.33±15 |
| Neoxanthin | 29.89±1.23*** | 13.84±2.82 |
| violaxanthin | 53.77±4.53 | 50.4±3.59 |
| zeaxanthin | 10.35±1.05*** | 5.31±0.38 |
| β-citraurin | 0.01±0 | 0.01±0 |
| total | 443.94±24.03 | 282.08±28.59 |

Table S6. The primers used in this study

| Primer name | Sequence | Purpose |
| --- | --- | --- |
| C05-40.45-F | TATTCAAAATTGGGTTTTACTG | Fina mapping |
| C05-40.45-R | GGCCCCAAAACCGGTTTCGT | Fina mapping  Fina mapping |
| C05-49.59-F | CTAGAGCCATCTTCACACA |  |
| C05-49.59-R | TTGCTTCTTGGCACTGTT | Fina mapping  Fina mapping |
| C05-50.01-F | TCTCTAATCTCCACTTGATC |  |
| C05-50.01-R | GCTCGGTTTAAGGTTAGT | Fina mapping  Fina mapping |
| 43.80-F | ATCGCTTCGGGTCGACTTAA |  |
| 43.80-R | CTCGAAAGTCGTCGAAAAC | Fina mapping  Fina mapping |
| 43.81-F | CAGCATGAGCAATTGGCTGA |  |
| 43.81-R | CGTCACTGCATAAAAGGCTC | Fina mapping  Fina mapping |
| 43.86-F | CTTGAGGGTTGTCAGAGAAT |  |
| 43.86-R | AACTCGTGAGCCGGTCTTA | Fina mapping  Fina mapping |
| 43.46-F | GAGCATGGAATATCCCTGTC |  |
| 43.46-R | AATAGGAACTCTGGGACCGGT | Fina mapping  Fina mapping |
| 43.74-F | CCACTTCTCCTCTAATAACC |  |
| 43.74-R | CGAGACGAAGAAGCGAAGTTG | Fina mapping  Fina mapping |
| 43.31-F | CTGACCCAAATTAAGACCGG |  |
| 43.31-R | GCGGAATATGAACGAGTGAAC | Fina mapping  Fina mapping |
| 42.06-F | AATGGATACTCGGAACCGAC |  |
| 42.06-R | CGAAAGACAACGGTTAAACCG | Fina mapping  RT-PCR |
| psbc-F | CTACCACGTGGAAACGCTCT |  |
| PSBC-R | CATTGCTCCGGCCCAGAATA | RT-PCR  RT-PCR |
| NDHH-F | CATGCACGGTGTTCTTCGCT |  |
| NDHH-R | TCCCACCGCGTTACATAAGG | RT-PCR  RT-PCR |
| RPOB-F | GACCGAGAAAGTGGAACTGC |  |
| RPOB-R | AACAGGCTGTACCAGCTCGA | RT-PCR  RT-PCR |
| PETB-F | TCAGTTCATCGATGGTCGGC |  |
| PETB-R | CCGGAATAGCGTCAGGTACA | RT-PCR  RT-PCR |
| MT-NAD6-F | GGTTTGATGGTTGCACGTGC |  |
| MT-NAD6-R | CTTCTTCGTGAATCTCCGCT | RT-PCR  RT-PCR |
| MT-ORF25-F | GACCACCAAGCTCTCTCGAA |  |
| MT-ORF25-R | TTACCAATGGCACGCTGTGC | RT-PCR  RT-PCR |
| MT-C0X3-F | GGTAGATCCAAGTCCATGGC |  |
| MT-C0X3-R | CGAGGTCCTAATTGTACGAC | RT-PCR  RT-PCR |
| MT-RPS7-F | GCGCGAAACTTCGATTGGTG |  |
| MT-RPS7-R | GCCAGGGATCGTCAACAAAC | RT-PCR  Complementarity |
| C05MUT-F | CGACTTTACAGAGGTCGCTG |  |
| C05MUT-R | TCATGGCAGTTCTCGAGTGG | Complementarity  Complementarity |
| PFGC541-C5POLIB-F | ATTTGAAAAATCTCAGAATTCGTTGCAGCCATGTGTCTTCA |  |
| PFGC541-C5POLIB-R | TTTAAATCATCGATTGGGCGCGCCCAAACCTGTTCGACATCGTC | Complementarity  Gene editing |
| DT1-BsF-C5POLIB | ATATATGGTCTCGATTGTCTTATTGTTGCTAACTATGTT |  |
| DT1-F0-C5POLIB | TGTCTTATTGTTGCTAACTATGTTTTAGAGCTAGAAATAGC | Gene editing  Gene editing |
| DT2-R0-C5POLIB | AACATAGTTAGCAACAATAAGACAATCTCTTAGTCGACTCTAC |  |
| DT2-BsR-C5POLIB | ATTATTGGTCTCGAAACATAGTTAGCAACAATAAGACAA | Gene editing |
