# Supplementary figures and images for: A single nucleotide mutation of BnaC05.POLIB creates yellow-white chimeric flower in Brassica napus
Source: Hortic Res. 2026 Jan 1;13(1):uhaf276. doi: 10.1093/hr/uhaf276 (PMC12903450; doi:10.1093/hr/uhaf276)

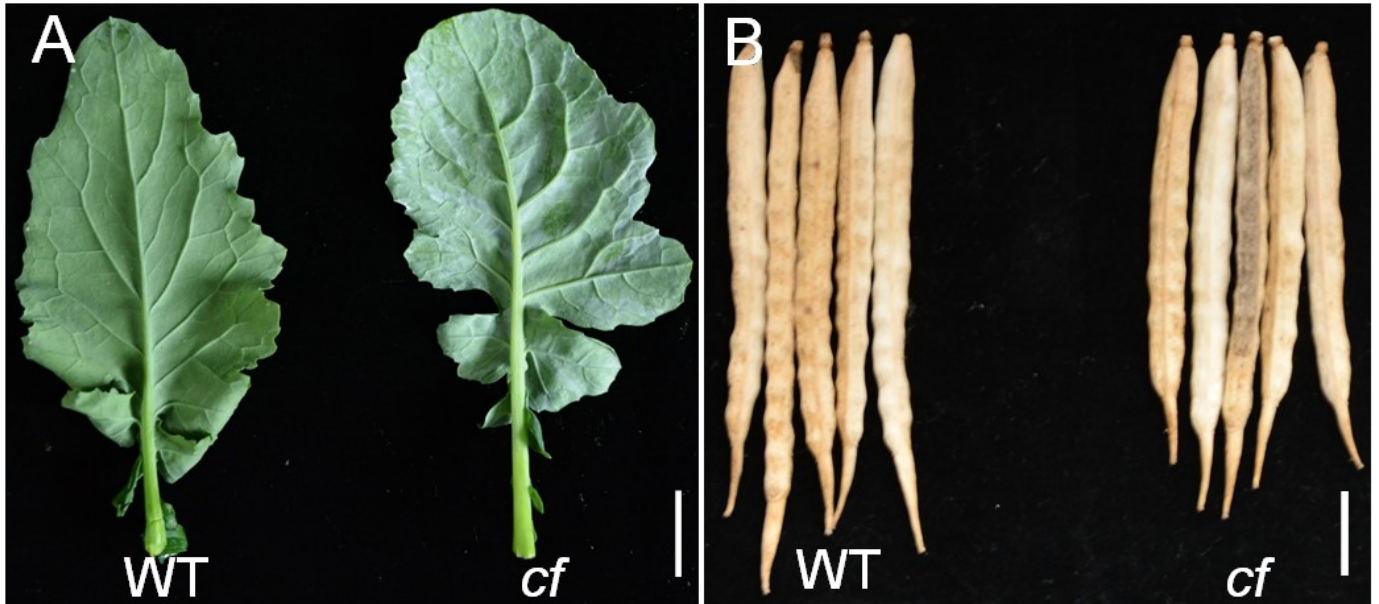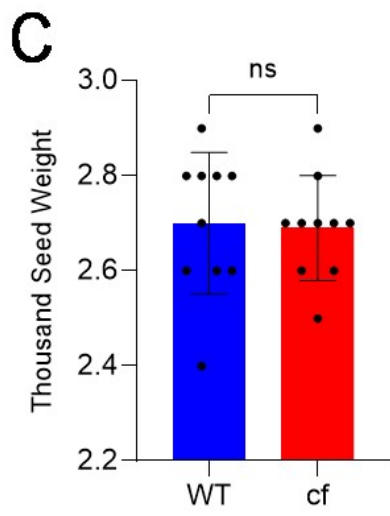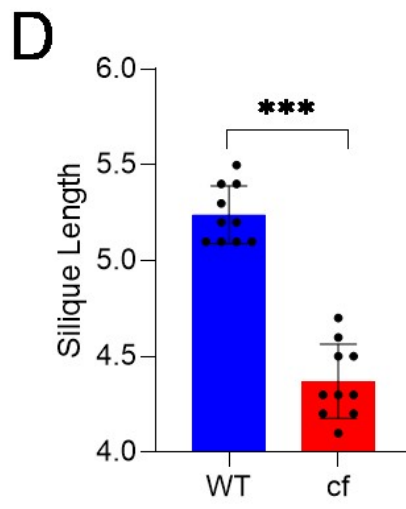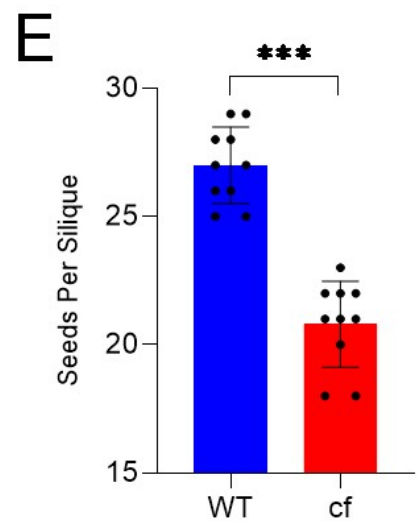

Supplement: Web_Material_uhaf276 [file web_material_uhaf276.zip › Supplementary Figure S1.pdf]

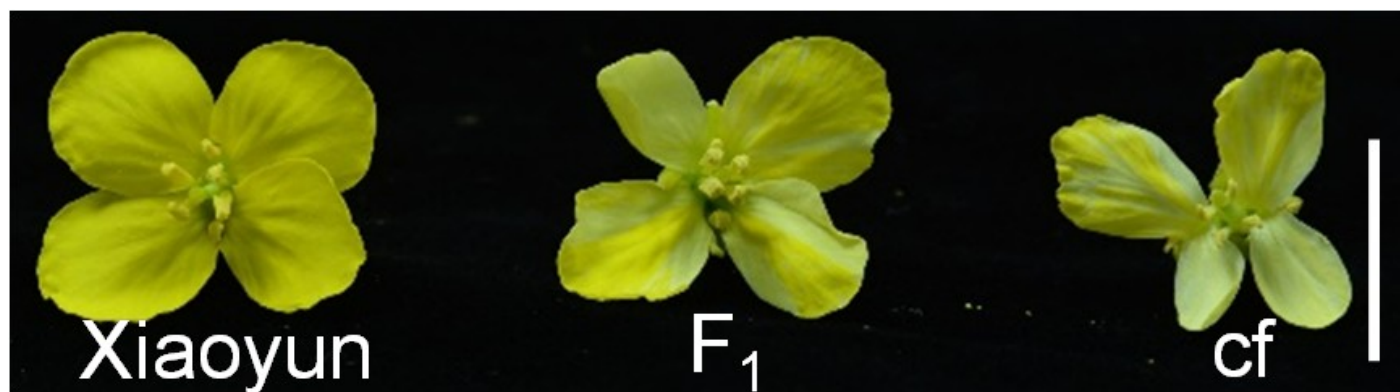

Supplement: Web_Material_uhaf276 [file web_material_uhaf276.zip › Supplementary Figure S2.pdf]

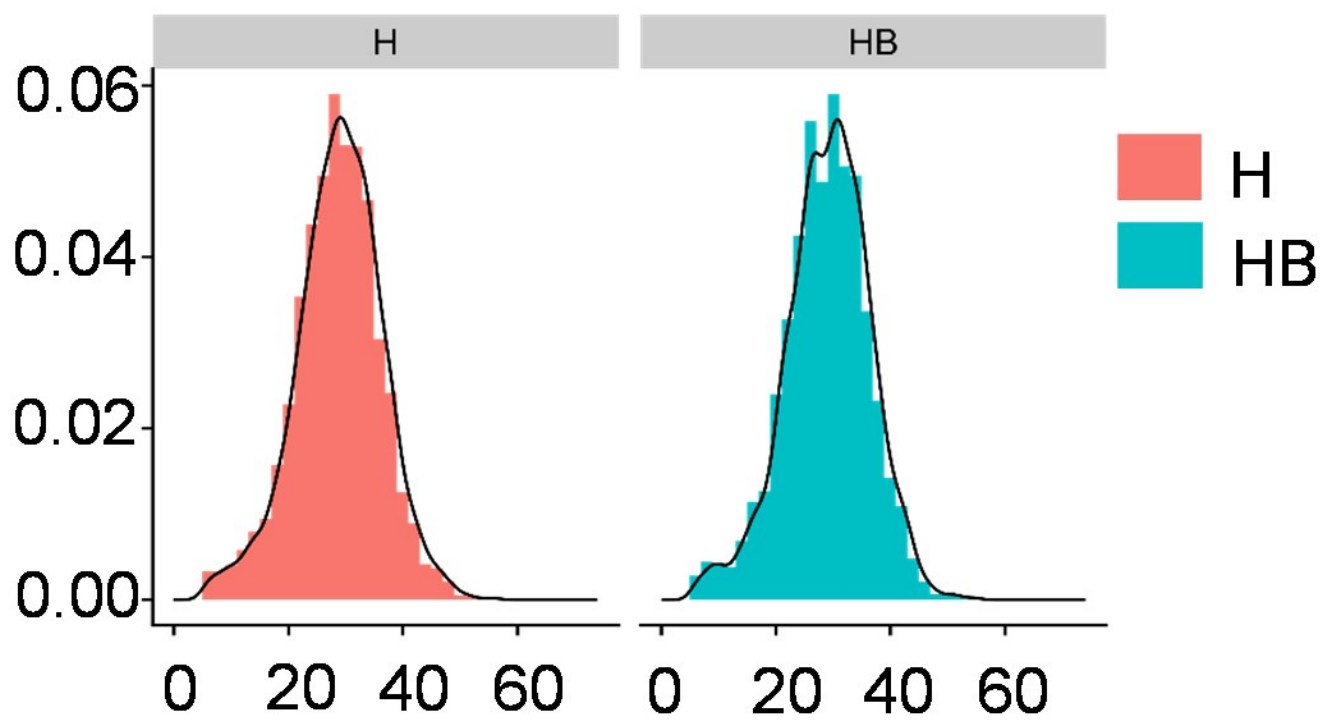

Supplement: Web_Material_uhaf276 [file web_material_uhaf276.zip › Supplementary Figure S3.pdf]

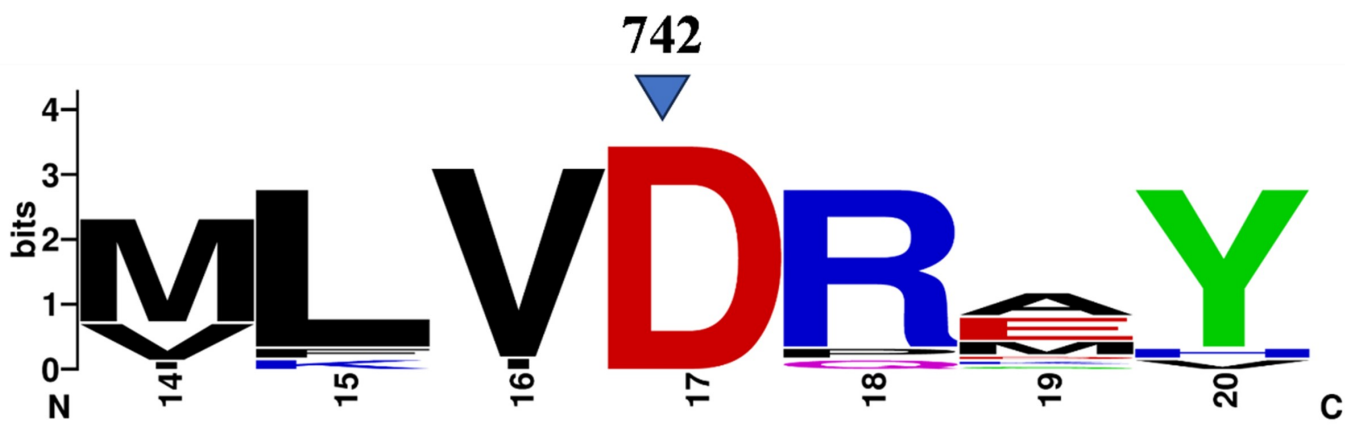

Supplement: Web_Material_uhaf276 [file web_material_uhaf276.zip › Supplementary Figure S5.pdf]

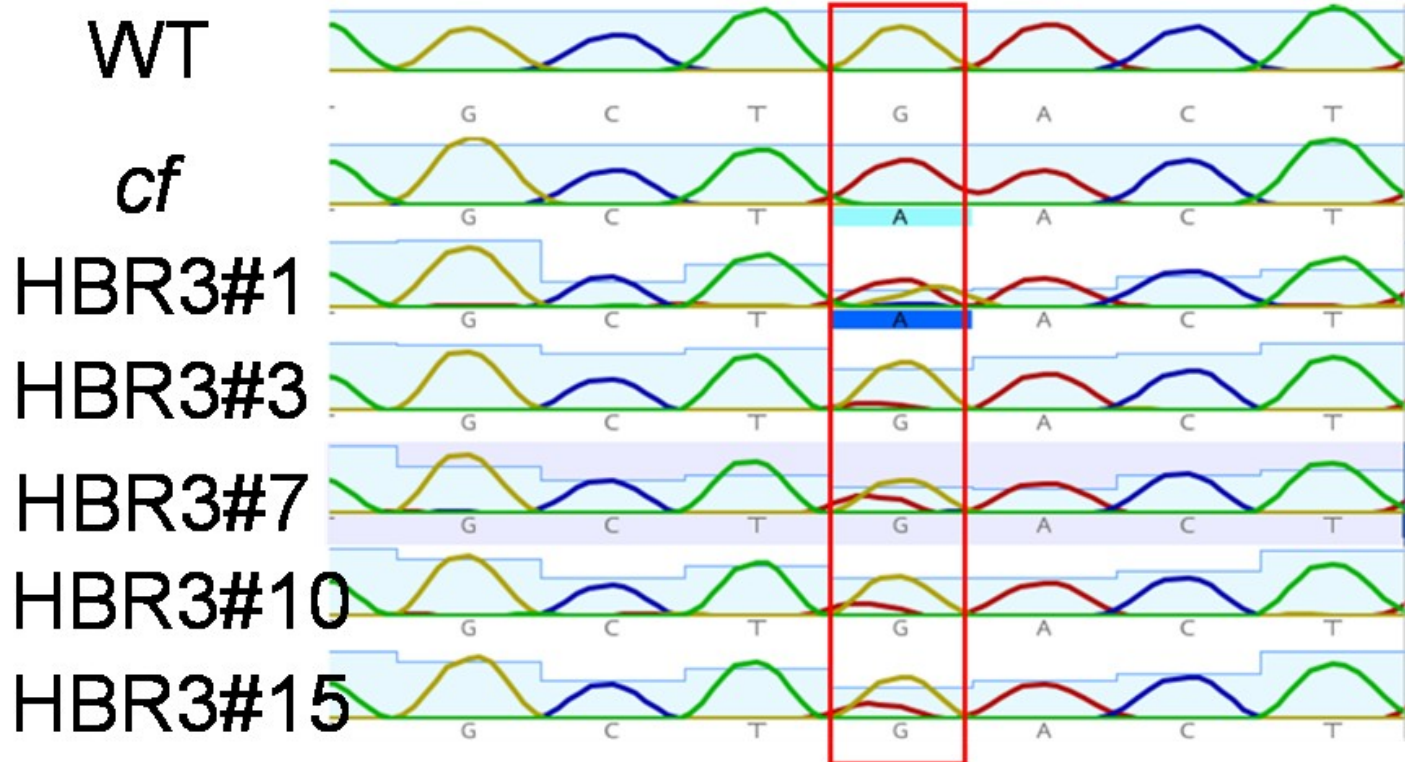

Supplement: Web_Material_uhaf276 [file web_material_uhaf276.zip › Supplementary Figure S6.pdf]

A

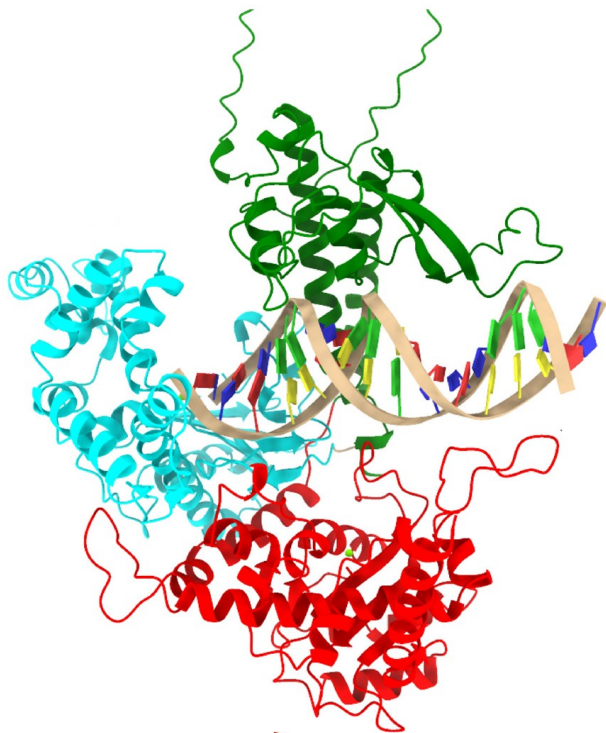

B

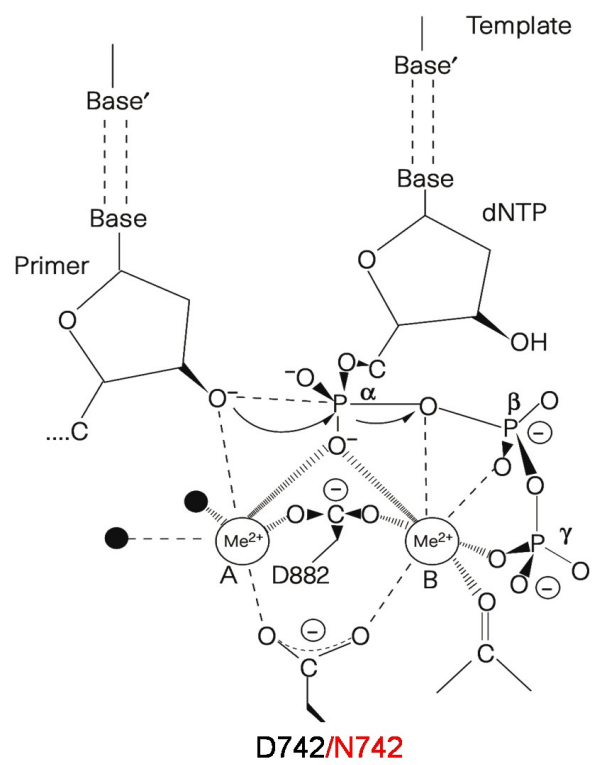

Supplement: Web_Material_uhaf276 [file web_material_uhaf276.zip › Supplementary Figure S7.pdf]

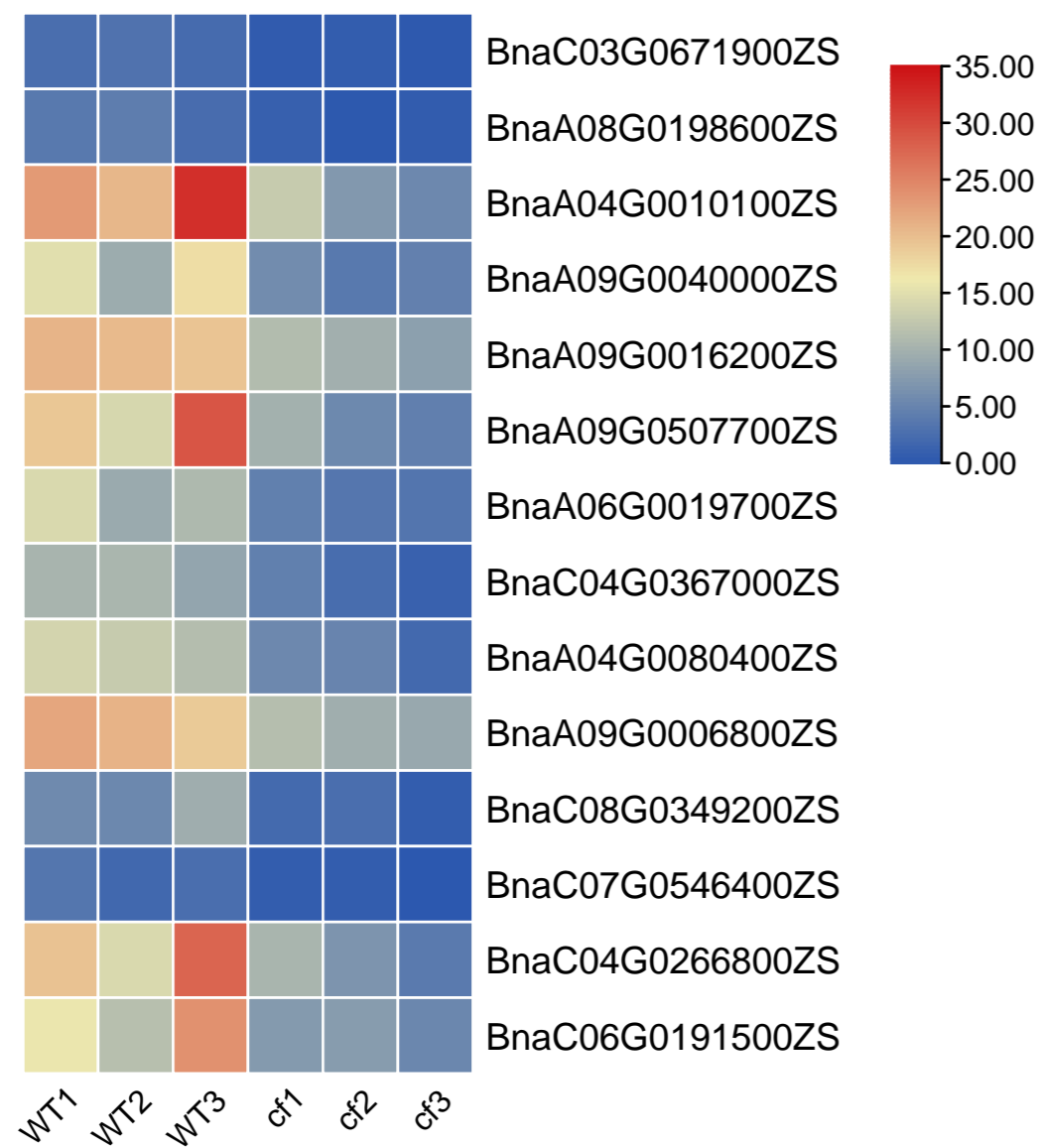

Supplement: Web_Material_uhaf276 [file web_material_uhaf276.zip › Supplementary Figure S8.pdf]

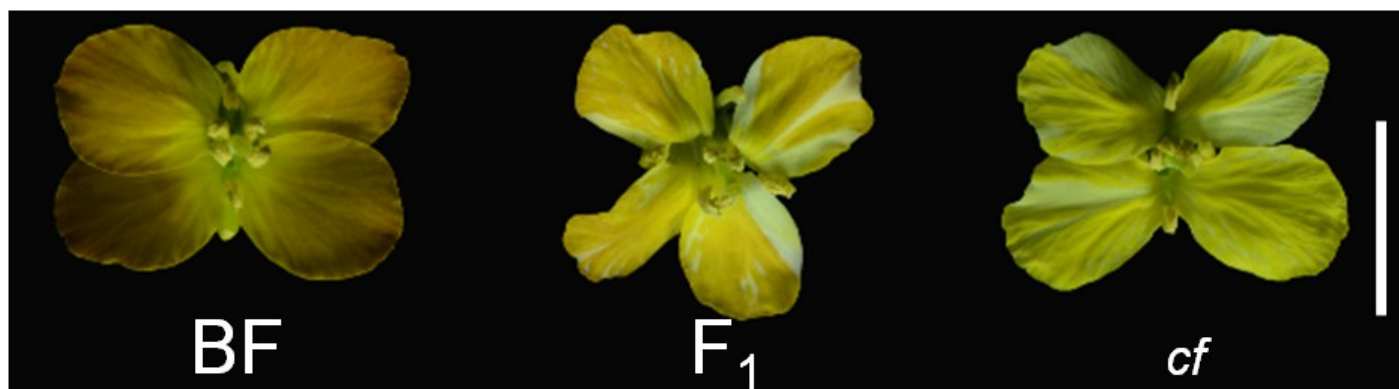

Supplement: Web_Material_uhaf276 [file web_material_uhaf276.zip › Supplementary Figure S9.pdf]

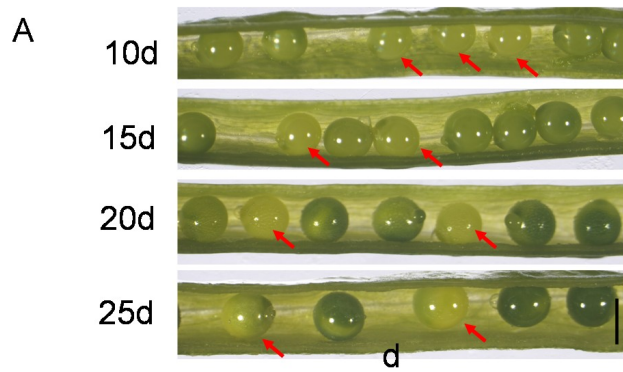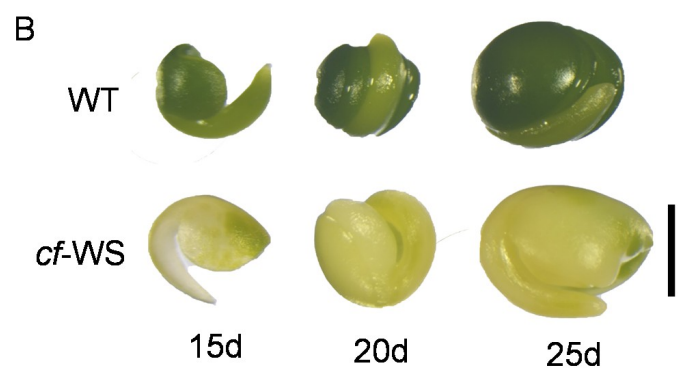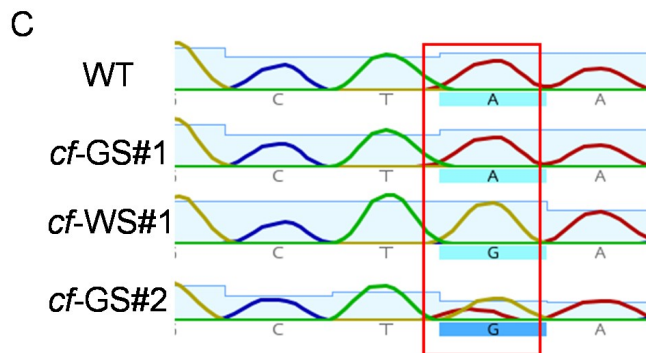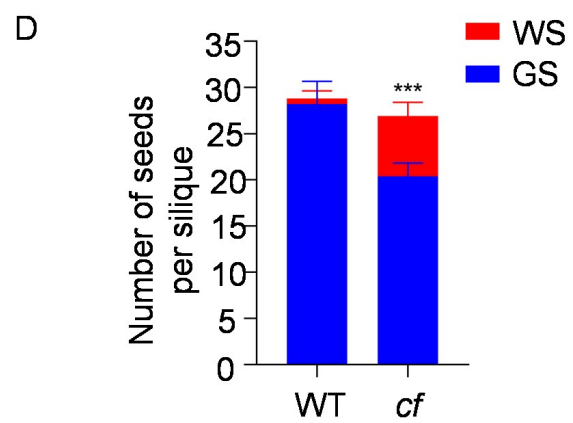

Supplement: Web_Material_uhaf276 [file web_material_uhaf276.zip › Supplementary Figure S10.pdf]
